# Genetic Diversity Relationship in Azakheli Buffalo Inferred from mtDNA and *MC1R* Sequences Comparison

**DOI:** 10.1155/2022/5770562

**Published:** 2022-12-13

**Authors:** Sikandar Ali, Fazul Nabi, Muhammad Awais, Syed Khurram Fareed, Johar Hussain, Adeniyi C. Adeola, Rajwali Khan, Nazeer Ahmed, Guobo Quan

**Affiliations:** ^1^Yunnan Animal Science and Veterinary Institute, Panlong County, Kunming, 650224 Yunnan Province, China; ^2^Dow Institute for Advanced Biological and Animal Research, Dow University of Health Sciences, Ojha Campus, Karachi 74200, Pakistan; ^3^Faculty of Veterinary and Animal Sciences, Labela University of Agriculture Water and Marine Sciences, Uthal, Pakistan; ^4^University of Health Sciences, Lahore, Pakistan; ^5^State Key Laboratory of Genetic Resources and Evolution, Kunming Institute of Zoology, Chinese Academy of Sciences, Kunming, 650223 Yunnan, China; ^6^Department of Livestock Management Breeding and Genetics, The University of Agriculture, Peshawar 25130, Pakistan

## Abstract

Azakheli is relatively smaller riverine breed with a very peculiar characteristics kept under unique traditional husbandry practices in comparison with rest of the Pakistani buffalo breeds; however, milk production is comparable. The present study was conducted to evaluate the genetic diversity of Azakheli breed. A total of sixty-six blood samples were collected for the amplification of mtDNA D-loop region and MC1R gene sequencing analysis. Median-joining network analysis of 191 mtDNA D-loop sequences of Azakheli and eight Indian riverine buffalo breeds clustered into three clades. Ancient Azakheli Region 1 clade was the oldest with the highest mutation steps and was present close to the root of UPGMA phylogenetic tree. There was 5 mutated lines distance between Pakistan buffalo and Indian riverine buffaloes. The populations of neighboring countries did not share any haplotypes with Azakheli buffalo of Pakistan. Possibly, residing for so long in the cold atmosphere and high elevation regions caused the mutation in mtDNA D-loop, though these conditions did not affect the overall performance of Azakheli as milch buffalo breed of Pakistan. MC1R analyses showed high mutations in Azakheli of Albino phenotype and all the black phenotype individuals of Azakheli buffalo share haplotypes with dominant Chinese and Indian black phenotypes buffaloes in MC1R median-joining network, indicating the reason of black coat color is due to MC1R gene. The haplotype diversity and nucleotide diversity was (H. 0.923, Pi: 0.00895) in Azakheli. Current results illustrated Asian ancestry for Azakheli buffalo, and mtDNA and MC1R analyses provided further evidence. Additional genetic analyses and archeological studies may provide further insight into the domestication period and history of Azakheli buffalo breed. The further studies are required on different coat colors with different genes on Azakheli buffalo to understand the phenotype variation.

## 1. Introduction

There are few countries in the world who produce buffalo milk, and most of them are Asiatic countries such as Pakistan, China, India, and Iran. Pakistan and India are enriched with milch type of buffaloes with tremendous potential in livestock sector for milk and meat production. Whereas, swamp buffalo breeds can be found in other countries, which are primarily used for transport, draught, and meat purposes in rural areas [1, 2]. The high buffalo milk-producing countries reared water buffalo (*Bubalus bubalis*) in tropical and subtropical regions as multipurpose agricultural animals. The marginal farmers and landless labourers of these regions mostly depend upon the products and by products of water buffaloes. Generally, water buffalo is further divided in to two types according to their, phenotype, genotype, and milk yield, i.e., riverine buffalo (2*n* = 50) distributed in Pakistan, Iran, Egypt, India, Middle East, and Eastern Europe. Swamp buffalo (2*n* = 48) is distributed in Thailand, Malaysia, China, Bangladesh, and the Southeast Asian countries. There is a total of five breeds of water buffaloes in Pakistan, including Nili, Ravi, Nili-Ravi, Kundi, and Azakheli [1–4]. Azakheli or Azikheli is an indigenous livestock dairy buffalo breed of Khyber Pakhtunkhwa (KP) province of Pakistan, also well known for its toughness, disease resistance, high fertility, survival on little inputs, and adaptability to variable environments [5, 6]. The farming pattern of Azakheli is only on small scale herds (1–10 animals/herd) mostly in cold, high altitude, and rural area. Azakheli buffalo was first found in Khawazakhela tribe and its surrounding areas in Swat District since unknown times. Azakheli buffalo requires lesser space and feed, due to relatively shorter height and smaller size as compare to other buffalo breeds of Pakistan; however, milk production is comparable. Therefore, Azakheli buffalo is raised under unique traditional husbandry practice. [7, 8]. [9–11] reported negative impact of cold weather on riverine buffalo breeds up to 30% decrease in milk production and other health issues. However, the Azakheli is the only riverine buffalo breed which is habituated in cold, snowy, and high-altitude regions of Pakistan. Overall performance of Azakheli buffalo is improving as compare to last decade, and the productivity is high as compare to Nilli-Ravi buffaloes present in the Swat District [5, 6]. Azakheli was first time recognized in 2006 census report as national breed of Pakistan with decline in population tendency [6, 7]. Such an indigenous breed with advantageous allele spectra acquired through adaptation to their environments could provide great economic stimulus for the future. The phylogeny and genetic diversity of water buffalo had been studied based on archeological and morphological evidences followed by the molecular method such as the mitochondrial DNA (mtDNA) polymorphism [12, 13]. Also, the examination of variation in D-loop region in elucidating the origin and diversification of modern buffalo populations has been previously reported [14]. Animal coloration is an ideal model for studying the genetic mechanisms that determine phenotype. Azakheli is characterized with relatively distinct color variation ranging from complete albino, brown, piebald, and to even black. The calves also have brownish and gray hair [9, 15]. The genetic polymorphism at (*melanocortin receptor 1*) *MC1R* gene has been investigated in some Chinese buffalo breeds [16], whereas several breeds of other domesticated animals, such as cattle, sheep, goat, horse, and pig have also been investigated [17]. *MC1R*, there is scanty study to show that the black coat coloration in Chinese buffalo is due to variation in *MC1R*. This include [16], but no research has been carried out on Azakheli breed of Pakistan. The domestic water-breed buffalo has not been given enough importance in terms of genetic improvement and evaluation [18]. According to [19] survey report, the Azakheli is better choice for buffalo farming in the cold mountainous region of KP province. Therefore, we used mtDNA and *MC1R* gene to investigate the genetic diversity and responsible gene for black coat color and SNPs of MC1R gene in Azakheli buffalo.

## 2. Materials and Methods

### 2.1. Sample Collection

**Figure 1:**
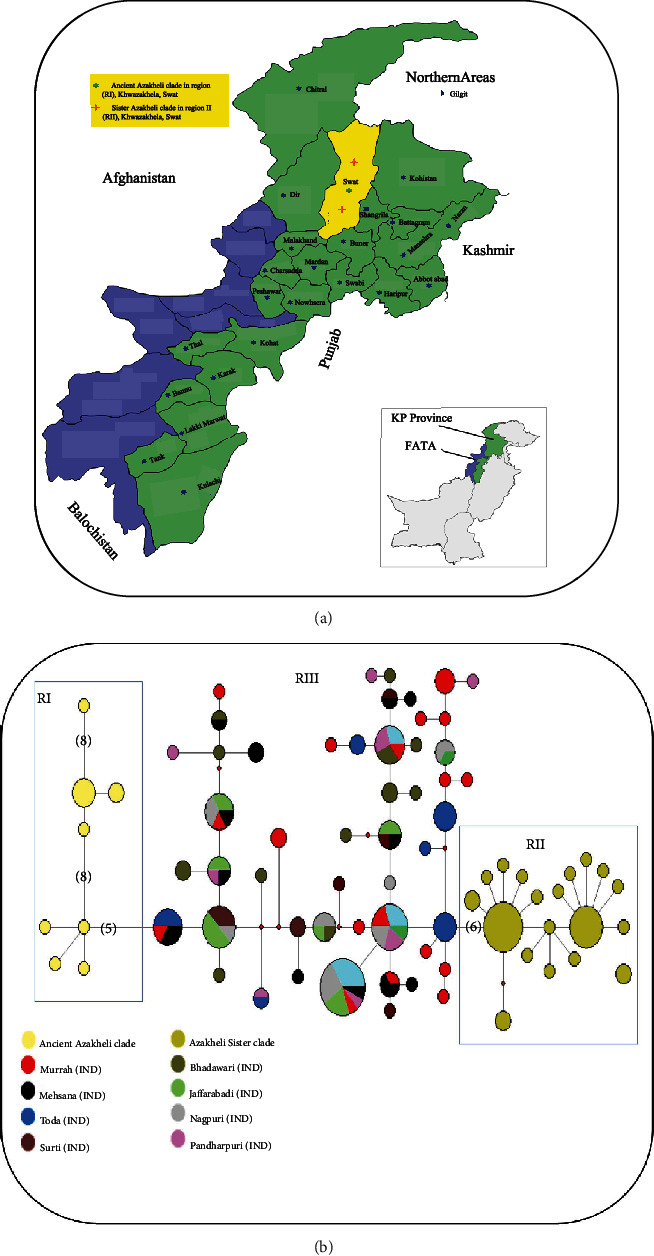
(a) Geographical indication of (Swat district) the study area in yellow color. (b) Median-joining network of 883 D-loop sequences corresponding to Pakistani Azakheli buffaloes. A circle represents every haplotype, and the area of the circle is proportionate to its frequency. Samples from different breeds are mentioned in different colors except Azakheli, which is shown in two different colors. The bracket () numbers in MJ haplotype network represents the highest numbers of mutated position lines in the network, and the haplotypes which have more mutated position lines are referred to as interior or ancestral haplotypes [31].

In this study, a total of 66 blood samples of Azakheli riverine buffaloes were collected from two regions, Region I (RI), Khawazakhela, Swat District and its surroundings Region II (RII) as shown in Figure 1(a). The details of each blood sample are present in (Supplementary Table S1). About 5 mL of fresh blood samples (EDTA as anticoagulant) were collected from the jugular vein of each Azakheli buffalo. Until genomic DNA extraction, all blood samples were stored at -20°C.

### 2.2. DNA Isolation, PCR, and MC1R Gene Amplification and Sequencing

The genomic DNA was isolated from these specimens using EasyPure Genomic DNA Isolation Kit (TransGen Biotech Co., Ltd, China) as per manufacturer's instructions, stored at -20°C. The control region in mtDNA D-loop was amplified to 883 bp segment using reference sequence (AF475214) and the Forward primer: (5′-TAGTGCTAATACCAACGGCC-3) and Reverse primer (5′-AGGCATTTTCAGTGCCTTGC-3). Primers were designed according to the sequence of MC1R gene using reference sequence (MF421415) primer and were divided into following parts: (Forward1 5′-CCAAGGAAGGCTCTGTTCTC-3′ reverse1 5′-CAGGATGACCTTGTGGTTGTAG-3′) and (Forward2 5′-GACCGCTACATCTCCATCTTCT-3′; reverse2 5′-TAGGTCTTACCCCTCTTCACCA-3′). The polymerase chain reaction mixture (50 *μ*L) contained 1 *μ*L of reverse primer (10 pmol/*μ*L), 1 *μ*L of forward primer (10 pmol/*μ*L), 22 *μ*L of ddH2O, 25*μ*L of premixed polymerase (TransGen Biotech Co., Ltd, China), and 1 *μ*L of DNA (50 ng/*μ*L). After denaturation at 95°C (5 mints), the PCR conditions were for 35 cycles at 95°C for 30 s, 62°C for 30 s, 72°C for 30 s, and a final extension at 72°C for 10 min. After collecting the PCR products, the quality was checked with 2% agarose gel electrophoresis then was purified and sequenced (TSINGKE, Bio. Tech. Beijing, PRC).

### 2.3. Data Analysis

As a result of DNA sequencing analysis, D-loop region of mtDNA sequences were aligned using ClustalW method and edited by the DNASTAR7.1 package (DNASTAR, Madison, WI). The evolutionary history of Azakheli was inferred using the UPGMA method, and the bootstrap consensus tree concluded from 1000 replicates by p-distance model using MEGAX [20]. Excluding all the gaps/missing data, the haplotype sequence data file was generated by using DnaSP version 5.10.01 [21]. Then, the median-joining (MJ) networks were constructed using the program Network 5.0.0.1 [22]. The sequence variation in MC1R was examined for 68 Azakheli by sequencing the entire 954 bp MC1R-coding region using PHASE version 21.1. [23, 24]. The study obtained 125 mtDNA control region sequences of different eight Indian riverine buffalo breeds from NCBI (see Supplementary Table S2).

## 3. Results and Discussion

### 3.1. The Analysis of mtDNA D-Loop Sequences

**Figure 2:**
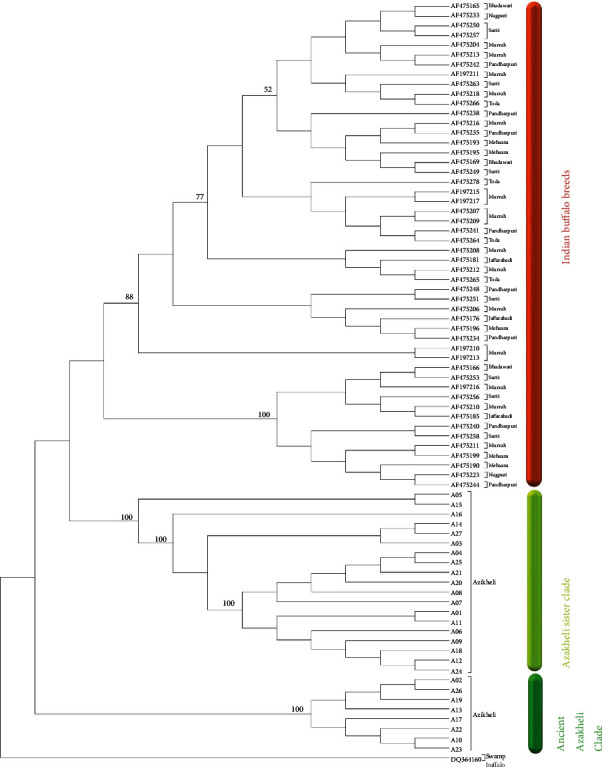
Rooted UPGMA phylogenetic tree based on mtDNA D-loop haplotypes. There was a total of 78 haplotypes with out-group (Access No: DQ364160) Chinese swamp buffalo as a base root. The haplotypes of (RI) were in the form of bunch close to the root of the phylogenetic tree that is why it is named as Ancient Azakheli clade. The haplotypes of (RII) clade were at upper branch of Ancient Azakheli clade branch in the form of group, which is named Sister Azakheli clade. The Indian buffalo breeds' haplotypes were at the top of phylogenetic tree as compared to Azakheli buffalo of Pakistan.

Previously, [16, 25] reported (503 bp) sequences of Azakheli buffalo while we provide (883 bp) on mtDNA control region sequences. Out of 191, mtDNA D-loop sequences of Azakheli and Indian buffalo individuals showed 77 haplotypes (see Supplementary Table S3). The MJ network of mtDNA control region sequences represented two different clades of Azakheli buffalo on the bases of regions and ancient time depth. (1) Ancient Azakheli clade: the blood samples of this clade were collected from (RI) Khwazakhela Swat. There are seven haplotypes in this clade from the total of 14 individuals of (RI), and in MJ network, it clustered away from Indian buffalo breeds by five mutation steps (Figure 1(b)). (2) Sister Azakheli clade: this clade has 19 haplotypes from 52 individuals of (RII) which were collected from surroundings of Swat District, except Khwazakhela. There are six mutation steps between the clade of Indian buffalo breeds and Sister Azakheli clade. Ancient Azakheli clade have two haplotypes (Azi13 and Azi26), which showed the highest time depth with eight mutational steps in each haplotype of (RI). While (RII) have only one (Azi15) haplotype with five mutational steps (Figure 1(b)). The details about UPGMA phylogenetic tree based on mtDNA D-loop haplotypes (Figure 2). According to the [26], the Azakheli and Indian riverine buffaloes shared the haplotypes with each other in the median-joining network of 492 bp mitochondrial D-loop sequences, whereas the population of Iranian riverine buffaloes did not share any haplotype with the population of (neighbour) Pakistani riverine buffaloes and as well as also with Indian riverine buffaloes. Likewise, [27] stated that Malagasy dung beetles living in the Mangoro drainage are only slightly differentiated at upstream localities on opposite sides of the riverine, but they did not share haplotypes and parallel outcomes can be seen in Malagasy reed frogs [28], a pattern congruent with our results. There was no sharing of haplotypes between Azakheli and Indian riverine buffaloes in median-joining network. The same results were reported by [11] in comparison study of Azakheli buffalo with swamp buffaloes. The climatic changes, high-elevated areas, and annual variations incur drastic effects on the genetic diversity of species [29]. Probably the genetic diversity of Azakheli was affected due to residing for so long in the cold weather and high-elevated habitat (Table 1). Azakheli is only riverine buffalo in Asia, performing in snowy mountainous regions and harsh conditions, whereas other Pakistani high-performing riverine buffaloes failed to perform [8, 11].

Previous studies showed that the genetic variation of modern population generally decreases with increasing distance from the domesticated center [30], whereas, in our study, genetic diversity increased with increasing distance between Indian buffalo breeds and Azakheli breed.

### 3.2. The Analysis of MC1R Gene

The *MC1R* analyses resulted in 22 haplotypes from 68, 198, and 142 sequences of Azakheli buffalo and Indian and Chinese buffaloes, respectively (see Supplementary Table S4). The 17 haplotypes of Azakheli were unique and ancient on as compared to other five haplotypes. The 268 individuals of haplotype AIC9 with black phenotype of Azakheli, Chinese, and Indian buffaloes showed a star-like shape indicative of population expansion (Figure 2).

**Figure 3:**
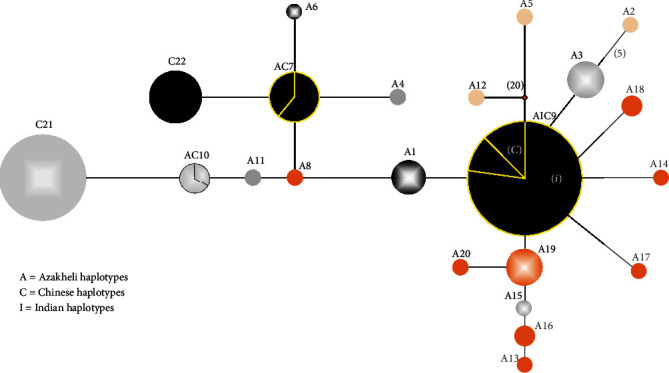
Median-joining network of MC1R haplotypes in Azakheli, Chinese, and Indian buffaloes. The circles areas are proportional to haplotype frequencies, and colors inside the circles denote the phenotypes of the buffaloes, such as A2, A5, and A12 represent Albino phenotype with highest mutation steps shown on the branch's length in () brackets. C21 and C22 haplotypes belong to Chinese buffaloes with grey-white and black phenotypes. AC10 and AC7 haplotypes were shared between Azakheli and Chinese buffaloes. A8 haplotype of Azakheli represents the brown color, and A19 represents brown color with white markings on the body of buffalo. A1 and A6 haplotypes of Azakheli denote the piebald color. Whereas, AIC9 denotes black phenotype shared between Azakheli, Indian, and Chinese buffaloes. The AC7 and C22 remaining unique haplotypes differed by a single synonymous substitution from AIC9 dominant black haplotypes.

All the individuals of Indian breeds were present in this haplotype with 73.9%, whereas 14.2% and 11.9% belonged to Chinese buffalo and Azakheli, respectively. All the black coat color individuals of Azakheli buffalo were present in AC7 and AIC9 haplotypes with other black coat color individuals of Chinese and Indian buffaloes (Figure 3). According to [16], the reason of black color phenotype in Chinese buffaloes is due to variation in *MC1R* gene. The MJ network of *MC1R* haplotypes revealed the signs and possibilities that black color phenotype of Azakheli buffalo is due to the variation of *MC1R* gene. The same results were reported [17] in Nigerian indigenous pigs, European wild type, and European dominant black and Asian dominant black haplotypes. To understand the mystery of different phenotypes in Azakheli buffalo, we suggest to analyze other coat color genes. The Haplotype diversity and nucleotide diversity were (H. 0.923, Pi: 0.00895) in Azakheli breed of Pakistan which was calculated as suggested by [21].

## 4. Conclusions

The current study concluded that existence of Azakheli breed is from ancient times and very unique riverine buffalo of Pakistan in terms of genetic material. In 883 bp sequences of Azakheli buffalo showed many mutations and SNPs, which led to conclusion of high genetic diversity and dissimilarities in mtDNA D-loop sequences with Indian riverine buffaloes. Probably, this changing occurred due to residing for so long in the cold weather and high elevation regions, which further studies need to carry. The MJ network of *MC1R* haplotypes revealed that black coat color of Azakheli buffalo is due to the *MC1R* gene same as in other black coat color of Indian and Chinese buffaloes. Further study needs to be conducted about different coat colors and pleiotropic effects of coat color genes. Our findings provide a valuable resource for future studies on whole-genome analyses of Azakheli buffalo.

## Figures and Tables

**Table 1:** The detail climatic and elevation information about the habitats of Azakheli and eight buffalo breeds of India.

S/no:	Breed & habitat	Min: max temp:	Elevation range	References
1	Azakheli, Sawat, Pakistan	-2°C to 33°C	516 to 3314 m	[5, 6]
2	Murrah, Haryana, India	8°C to 42°C	200 to 1200 m	http://www.weather-and-climate.com
3	Bhadawari, Uttar Pardesh, India	10°C to 43°C	200 to 237 m	http://www.weather-and-climate.com
4	Surti, Gujarat, India	12°C to 41°C	100 to 150 m	http://www.weather-and-climate.com
5	Jaffarabadi, Gujarat, India	12°C to 41°C	100 to 150 m	http://www.weather-and-climate.com
6	Mehsana, Gujarat, India	12°C to 41°C	100 to 150 m	http://www.weather-and-climate.com
7	Nagpuri, Maharastra, India	23°C to 32°C	1100 to 1200 m	http://www.weather-and-climate.com
8	Pandharpuri, Maharastra, India	23°C to 32°C	1100 to 1200 m	http://www.weather-and-climate.com
9	Toda, Tamil Nadu, India	25°C to 33°C	6 to 60 m	http://www.weather-and-climate.com

## Data Availability

The MC1R gene sequencing data used to support the findings of this study have not been made available because of further use. The mtDNA D-loop sequencing data used to support the findings of this study are available from the corresponding author upon request.

## Supplementary Materials

Supplementary 1 Supplementary Table S1: details on 66 blood samples of Azakheli buffalo.

Supplementary 2 Supplementary Table S2: the data of 125 Indian buffaloes mitochondrial control region sequences retrieved from GenBank were used for the median-joining network analysis Figure 1(b).

Supplementary 3 Supplementary Table S3: this table is showing 27 haplotypes from 66 blood samples of Azakheli collected from KP, Pakistan and 50 haplotypes from 125 mt-DNA sequences downloaded from NCBI. Hap No represents the total number of haplotypes; Hap name column shows the name of each haplotype. Different numbers in columns shows the SNP on the sequence position. T. NO column expresses the strength of samples per haplotypes. Dots (.) denote identity with the reference sequence. Short line (-) represents the absence of one haplotype from a certain breed. Whereas, rest of the column represents the names of buffalo breeds, i.e., (AZI: Azakheli; MUR: Murrah; BHA: Bhadawari; JAF: Jaffarabadi; MEH: Mehsana; NAG: Nagpuri; PAN: Pandharpuri; SUR: Surti; TAD: Toda.

Supplementary 4 Supplementary Table S4: data on 68 sequences of Azakheli buffalo of *MC1R* haplotypes including 198 Indian buffaloes and 142 Chinese buffalo sequences. The phenotype of all individuals was defined in the table. Chinese and Indian buffaloes *MC1R* haplotype sequences retrieved from GenBank were used for the median-joining network analysis (Figure 3).
