# Supplementary material for: Genetic Diversity Relationship in Azakheli Buffalo Inferred from mtDNA and MC1R Sequences Comparison
Source: Biomed Res Int. 2022 Dec 13;2022:5770562. doi: 10.1155/2022/5770562 (PMC9806686; doi:10.1155/2022/5770562)
Supplement: Supplementary 1 — Supplementary Table S1: details on 66 blood samples of Azakheli buffalo. [file 5770562.f1.docx]

**Supplementary Table S1.** Details on 66 blood samples of Azakheli buffalo.

| S/No | Sample No | Blood amount | Location | S/No | Sample No | Blood amount | Location |
| --- | --- | --- | --- | --- | --- | --- | --- |
| 1 | Azi 272 Azikheli | 5ml | RI | 34 | Azi 308 Azikheli | 5ml | RII |
| 2 | Azi 273 Azikheli | 5ml | RI | 35 | Azi 309 Azikheli | 5ml | RII |
| 3 | Azi 274 Azikheli | 5ml | RI | 36 | Azi 310 Azikheli | 5ml | RII |
| 4 | Azi 275 Azikheli | 5ml | RI | 37 | Azi 311 Azikheli | 5ml | RII |
| 5 | Azi 276 Azikheli | 5ml | RI | 38 | Azi 312 Azikheli | 5ml | RII |
| 6 | Azi 277 Azikheli | 5ml | RI | 39 | Azi 313 Azikheli | 5ml | RII |
| 7 | Azi 278 Azikheli | 5ml | RI | 40 | Azi 314 Azikheli | 5ml | RII |
| 8 | Azi 279 Azikheli | 5ml | RI | 41 | Azi 315 Azikheli | 5ml | RII |
| 9 | Azi 280 Azikheli | 5ml | RI | 42 | Azi 316 Azikheli | 5ml | RII |
| 10 | Azi 281 Azikheli | 5ml | RI | 43 | Azi 317 Azikheli | 5ml | RII |
| 11 | Azi 282 Azikheli | 5ml | RII | 44 | Azi 318 Azikheli | 5ml | RII |
| 12 | Azi 283 Azikheli | 5ml | RII | 45 | Azi 319 Azikheli | 5ml | RII |
| 13 | Azi 284 Azikheli | 5ml | RII | 46 | Azi 320 Azikheli | 5ml | RII |
| 14 | Azi 285 Azikheli | 5ml | RII | 47 | Azi 321 Azikheli | 5ml | RII |
| 15 | Azi 286 Azikheli | 5ml | RII | 48 | Azi 322 Azikheli | 5ml | RII |
| 16 | Azi 287 Azikheli | 5ml | RII | 49 | Azi 323 Azikheli | 5ml | RII |
| 17 | Azi 288 Azikheli | 5ml | RII | 50 | Azi 324 Azikheli | 5ml | RII |
| 18 | Azi 289 Azikheli | 5ml | RII | 51 | Azi 325 Azikheli | 5ml | RII |
| 19 | Azi 290 Azikheli | 5ml | RII | 52 | Azi 326 Azikheli | 5ml | RII |
| 20 | Azi 291 Azikheli | 5ml | RII | 53 | Azi 327 Azikheli | 5ml | RII |
| 21 | Azi 292 Azikheli | 5ml | RII | 54 | Azi 328 Azikheli | 5ml | RII |
| 22 | Azi 293 Azikheli | 5ml | RII | 55 | Azi 329 Azikheli | 5ml | RII |
| 23 | Azi 294 Azikheli | 5ml | RII | 56 | Azi 330 Azikheli | 5ml | RII |
| 24 | Azi 295 Azikheli | 5ml | RII | 57 | Azi 331 Azikheli | 5ml | RII |
| 25 | Azi 296 Azikheli | 5ml | RII | 58 | Azi 336 Azikheli | 5ml | RII |
| 26 | Azi 297 Azikheli | 5ml | RII | 59 | Azi 337 Azikheli | 5ml | RII |
| 27 | Azi 298 Azikheli | 5ml | RII | 60 | Azi 342 Azikheli | 5ml | RII |
| 28 | Azi 299 Azikheli | 5ml | RII | 61 | Azi 343 Azikheli | 5ml | RII |
| 29 | Azi 300 Azikheli | 5ml | RI | 62 | Azi 344 Azikheli | 5ml | RII |
| 30 | Azi 301 Azikheli | 5ml | RI | 63 | Azi 345 Azikheli | 5ml | RII |
| 31 | Azi 304 Azikheli | 5ml | RI | 64 | Azi 346 Azikheli | 5ml | RII |
| 32 | Azi 305 Azikheli | 5ml | RI | 65 | Azi 347 Azikheli | 5ml | RII |
| 33 | Azi 306 Azikheli | 5ml | RII | 66 | Azi 348 Azikheli | 5ml | RII |

Note: RI region represents the KHWAZAKHELLA region of SWAT.

RII region represents the surrounding areas of SWAT.
