# Supplementary material for: Genetic Diversity Relationship in Azakheli Buffalo Inferred from mtDNA and MC1R Sequences Comparison
Source: Biomed Res Int. 2022 Dec 13;2022:5770562. doi: 10.1155/2022/5770562 (PMC9806686; doi:10.1155/2022/5770562)
Supplement: Supplementary 2 — Supplementary Table S2: the data of 125 Indian buffaloes mitochondrial control region sequences retrieved from GenBank were used for the median-joining network analysis Figure 1(b). [file 5770562.f2.docx]

**Supplementary Table S2.** The data of 125 Indian Buffaloes mitochondrial control region sequences retrieved from GenBank were used for the median joining network analysis Figure 1B.

| **S/**  **No** | **Accession No:** | **Breed name** | **Location** | **S/**  **No** | **Accession No:** | **Breed name** | **Location** |
| --- | --- | --- | --- | --- | --- | --- | --- |
| 1 | AF475278 | Toda | Tamil Nadu | 64 | AF475213 | Murrah | Haryana |
| 2 | AF475277 | Toda | Tamil Nadu | 65 | AF475212 | Murrah | Haryana |
| 3 | AF475276 | Toda | Tamil Nadu | 66 | AF475211 | Murrah | Haryana |
| 4 | AF475275 | Toda | Tamil Nadu | 67 | AF475210 | Murrah | Haryana |
| 5 | AF475274 | Toda | Tamil Nadu | 68 | AF475209 | Murrah | Haryana |
| 6 | AF475273 | Toda | Tamil Nadu | 69 | AF475208 | Murrah | Haryana |
| 7 | AF475272 | Toda | Tamil Nadu | 70 | AF475207 | Murrah | Haryana |
| 8 | AF475271 | Toda | Tamil Nadu | 71 | AF475206 | Murrah | Haryana |
| 9 | AF475270 | Toda | Tamil Nadu | 72 | AF475205 | Murrah | Haryana |
| 10 | AF475269 | Toda | Tamil Nadu | 73 | AF475204 | Murrah | Haryana |
| 11 | AF475268 | Toda | Tamil Nadu | 74 | AF475203 | Mehsana | Gujarat |
| 12 | AF475267 | Toda | Tamil Nadu | 75 | AF475202 | Mehsana | Gujarat |
| 13 | AF475266 | Toda | Tamil Nadu | 76 | AF475201 | Mehsana | Gujarat |
| 14 | AF475265 | Toda | Tamil Nadu | 77 | AF475200 | Mehsana | Gujarat |
| 15 | AF475264 | Toda | Tamil Nadu | 78 | AF475199 | Mehsana | Gujarat |
| 16 | AF475263 | Surti | Gujarat | 79 | AF475198 | Mehsana | Gujarat |
| 17 | AF475262 | Surti | Gujarat | 80 | AF475197 | Mehsana | Gujarat |
| 18 | AF475261 | Surti | Gujarat | 81 | AF475196 | Mehsana | Gujarat |
| 19 | AF475260 | Surti | Gujarat | 82 | AF475195 | Mehsana | Gujarat |
| 20 | AF475258 | Surti | Gujarat | 83 | AF475194 | Mehsana | Gujarat |
| 21 | AF475257 | Surti | Gujarat | 84 | AF475193 | Mehsana | Gujarat |
| 22 | AF475255 | Surti | Gujarat | 85 | AF475192 | Mehsana | Gujarat |
| 23 | AF475254 | Surti | Gujarat | 86 | AF475191 | Mehsana | Gujarat |
| 24 | AF475253 | Surti | Gujarat | 87 | AF475190 | Mehsana | Gujarat |
| 25 | AF475252 | Surti | Gujarat | 88 | AF475189 | Mehsana | Gujarat |
| 26 | AF475251 | Surti | Gujarat | 89 | AF475188 | Jaffarabdi | Gujarat |
| 27 | AF475250 | Surti | Gujarat | 90 | AF475186 | Jaffarabdi | Gujarat |
| 28 | AF475249 | Surti | Gujarat | 91 | AF475186 | Jaffarabdi | Gujarat |
| 29 | AF475248 | Pandharpuri | Maharastra | 92 | AF475185 | Jaffarabdi | Gujarat |
| 30 | AF475247 | Pandharpuri | Maharastra | 93 | AF475184 | Jaffarabdi | Gujarat |
| 31 | AF475246 | Pandharpuri | Maharastra | 94 | AF475183 | Jaffarabdi | Gujarat |
| 32 | AF475245 | Pandharpuri | Maharastra | 95 | AF475182 | Jaffarabdi | Gujarat |
| 33 | AF475244 | Pandharpuri | Maharastra | 96 | AF475181 | Jaffarabdi | Gujarat |
| 34 | AF475243 | Pandharpuri | Maharastra | 97 | AF475180 | Jaffarabdi | Gujarat |
| 35 | AF475242 | Pandharpuri | Maharastra | 98 | AF475179 | Jaffarabdi | Gujarat |
| 36 | AF475241 | Pandharpuri | Maharastra | 99 | AF475178 | Jaffarabdi | Gujarat |
| 37 | AF475241 | Pandharpuri | Maharastra | 100 | AF475177 | Jaffarabdi | Gujarat |
| 38 | AF475239 | Pandharpuri | Maharastra | 101 | AF475176 | Jaffarabdi | Gujarat |
| 39 | AF475238 | Pandharpuri | Maharastra | 102 | AF475175 | Jaffarabdi | Gujarat |
| 40 | AF475237 | Pandharpuri | Maharastra | 103 | AF475174 | Jaffarabdi | Gujarat |
| 41 | AF475236 | Pandharpuri | Maharastra | 104 | AF475173 | Bhadawari | Uttar Pradesh |
| 42 | AF475235 | Pandharpuri | Maharastra | 105 | AF475172 | Bhadawari | Uttar Pradesh |
| 43 | AF475234 | Pandharpuri | Maharastra | 106 | AF475171 | Bhadawari | Uttar Pradesh |
| 44 | AF475233 | Nagpuri | Maharastra | 107 | AF475170 | Bhadawari | Uttar Pradesh |
| 45 | AF475232 | Nagpuri | Maharastra | 108 | AF475169 | Bhadawari | Uttar Pradesh |
| 46 | AF475231 | Nagpuri | Maharastra | 109 | AF475168 | Bhadawari | Uttar Pradesh |
| 47 | AF475230 | Nagpuri | Maharastra | 110 | AF475167 | Bhadawari | Uttar Pradesh |
| 48 | AF475229 | Nagpuri | Maharastra | 111 | AF475166 | Bhadawari | Uttar Pradesh |
| 49 | AF475228 | Nagpuri | Maharastra | 112 | AF475165 | Bhadawari | Uttar Pradesh |
| 50 | AF475227 | Nagpuri | Maharastra | 113 | AF475164 | Bhadawari | Uttar Pradesh |
| 51 | AF475226 | Nagpuri | Maharastra | 114 | AF475163 | Bhadawari | Uttar Pradesh |
| 52 | AF475225 | Nagpuri | Maharastra | 115 | AF475162 | Bhadawari | Uttar Pradesh |
| 53 | AF475224 | Nagpuri | Maharastra | 116 | AF475161 | Bhadawari | Uttar Pradesh |
| 54 | AF475223 | Nagpuri | Maharastra | 117 | AF475160 | Bhadawari | Uttar Pradesh |
| 55 | AF475222 | Nagpuri | Maharastra | 118 | AF475159 | Bhadawari | Uttar Pradesh |
| 56 | AF475221 | Nagpuri | Maharastra | 119 | AF197217 | Murrah | Haryana |
| 57 | AF475220 | Nagpuri | Maharastra | 120 | AF197216 | Murrah | Haryana |
| 58 | AF475219 | Nagpuri | Maharastra | 121 | AF197215 | Murrah | Haryana |
| 59 | AF475218 | Murrah | Haryana | 122 | AF197213 | Murrah | Haryana |
| 60 | AF475217 | Murrah | Haryana | 123 | AF197211 | Murrah | Haryana |
| 61 | AF475216 | Murrah | Haryana | 124 | AF197210 | Murrah | Haryana |
| 62 | AF475215 | Murrah | Haryana | 125 | AF197209 | Murrah | Haryana |
| 63 | AF475214 | Murrah | Haryana |  |  |  |  |
