# Supplementary material for: Genetic Diversity Relationship in Azakheli Buffalo Inferred from mtDNA and MC1R Sequences Comparison
Source: Biomed Res Int. 2022 Dec 13;2022:5770562. doi: 10.1155/2022/5770562 (PMC9806686; doi:10.1155/2022/5770562)
Supplement: Supplementary 3 — Supplementary Table S3: this table is showing 27 haplotypes from 66 blood samples of Azakheli collected from KP, Pakistan and 50 haplotypes from 125 mt-DNA sequences downloaded from NCBI. Hap No represents the total number of haplotypes; Hap name column shows the name of each haplotype. Different numbers in columns shows the SNP on the sequence position. T. NO column expresses the strength of samples per haplotypes. Dots (.) denote identity with the reference sequence. Short line (-) represents the absence of one haplotype from a certain breed. Whereas, rest of the column represents the names of buffalo breeds, i.e., (AZI: Azakheli; MUR: Murrah; BHA: Bhadawari; JAF: Jaffarabadi; MEH: Mehsana; NAG: Nagpuri; PAN: Pandharpuri; SUR: Surti; TAD: Toda. [file 5770562.f3.docx]

**Supplementary Table S3.** This table is showing 27 haplotypes from 66 blood samples of Azakheli collected from KPK, Pakistan and 50 haplotypes from 125 mt-DNA sequences downloaded from NCBI. Hap: No: represents the total number of haplotypes, Hap: name column shows the name of each haplotype. Different Numbers in columns shows the SNP on the sequence position. T. NO column expresses the strength of samples per haplotypes. Dots (.) denote identity with the reference sequence. Short line (-) represents the absence of one haplotype from a certain breed. Whereas, rest of the column represents the names of buffalo breeds i.e. (AZI= Azakheli, MUR= Murrah, BHA= Bhadawari, JAF= Jaffarabadi, MEH= Mehsana, NAG= Nagpuri, PAN= Pandharpuri, SUR= Surti, and TAD= Toda.

| **Hap**  **No:** | **Hap:**  **Name** | **1**  **2**  **1** | **1**  **7**  **8** | **1**  **9**  **0** | **1**  **9**  **1** | **1**  **9**  **8** | **2**  **0**  **0** | **2**  **2**  **0** | **2**  **4**  **7** | **2**  **4**  **9** | **2**  **6**  **6** | **2**  **8**  **2** | **2**  **8**  **3** | **2**  **8**  **4** | **2**  **8**  **9** | **2**  **9**  **5** | **3**  **1**  **0** | **3**  **1**  **8** | **3**  **2**  **1** | **3**  **2**  **8** | **3**  **3**  **6** | **3**  **4**  **7** | **3**  **5**  **1** | **3**  **5**  **3** | **3**  **5**  **4** | **3**  **5**  **5** | **3**  **7**  **1** | **3**  **7**  **6** | **3**  **7**  **9** | **3**  **9**  **4** | **4**  **1**  **1** | **4**  **2**  **5** | **4**  **2**  **9** | **4**  **5**  **7** | **4**  **5**  **8** | **4**  **5**  **9** |
| --- | --- | --- | --- | --- | --- | --- | --- | --- | --- | --- | --- | --- | --- | --- | --- | --- | --- | --- | --- | --- | --- | --- | --- | --- | --- | --- | --- | --- | --- | --- | --- | --- | --- | --- | --- | --- |
| **1** | **A01** | G | T | G | A | C | C | C | T | C | G | A | A | A | C | A | T | G | C | A | T | C | C | C | A | T | T | T | G | C | T | G | G | G | T | C |
| **2** | **A02** | C | . | A | . | A | T | T | . | . | . | G | . | . | . | G | . | . | . | . | . | . | . | T | G | . | . | C | . | . | . | . | . | . | . | . |
| **3** | **A03** | . | . | . | . | A | . | T | . | . | . | . | . | . | . | . | . | A | . | . | . | . | . | . | . | . | . | . | . | . | . | . | . | . | . | . |
| **4** | **A04** | . | . | . | . | A | T | . | . | . | . | . | . | . | . | . | . | . | . | . | . | . | . | . | . | . | . | . | . | . | . | . | . | . | . | . |
| **5** | **A05** | . | . | . | . | G | T | . | . | . | . | . | G | . | . | . | . | . | . | . | . | . | . | T | . | C | C | C | . | . | . | . | . | . | . | . |
| **6** | **A06** | . | . | . | . | A | T | . | . | . | . | . | . | . | . | . | . | . | . | . | . | . | . | . | . | . | . | . | . | . | . | . | . | . | . | . |
| **7** | **A07** | . | . | . | . | A | T | . | . | . | . | . | . | . | . | . | . | . | . | . | . | . | . | . | . | . | . | . | . | . | . | . | . | . | . | . |
| **8** | **A08** | . | . | . | . | A | T | . | . | . | . | . | . | . | . | . | . | . | . | . | C | . | . | . | . | . | . | . | . | . | . | . | . | . | . | . |
| **9** | **A09** | . | . | . | . | A | T | . | . | . | . | . | . | . | . | . | . | . | T | . | . | . | . | . | . | . | . | . | . | . | . | . | . | . | . | . |
| **10** | **A10** | . | . | . | . | A | T | . | . | . | . | . | . | . | . | . | . | . | . | . | . | . | . | T | . | . | . | C | . | . | C | . | A | . | . | T |
| **11** | **A11** | . | . | . | . | A | . | . | . | . | . | . | . | . | . | . | . | . | . | . | . | T | . | . | . | . | . | . | . | . | . | . | . | . | . | . |
| **12** | **A12** | . | . | . | . | A | T | . | . | . | . | . | . | . | . | . | . | . | . | . | . | . | T | . | . | . | . | . | . | . | . | . | . | . | . | . |
| **13** | **A13** | . | . | A | G | A | T | T | C | . | . | G | . | . | . | . | . | . | . | . | . | . | . | T | . | C | . | C | . | T | . | . | . | . | C | . |
| **14** | **A14** | . | . | . | . | A | . | . | . | . | . | . | . | G | . | . | . | . | . | G | . | . | . | . | . | . | . | . | . | . | . | . | . | . | . | . |
| **15** | **A15** | . | . | . | . | G | T | . | . | T | . | . | G | . | . | . | . | . | . | . | . | . | . | T | . | . | . | . | . | . | . | . | . | . | . | . |
| **16** | **A16** | . | . | . | . | A | . | . | . | . | . | G | . | . | . | . | . | . | . | . | . | . | . | . | . | . | . | . | . | . | . | . | . | . | . | . |
| **17** | **A17** | . | . | . | . | A | T | . | . | . | . | . | . | . | . | . | C | . | . | . | . | . | . | T | . | . | . | C | A | . | C | . | A | . | . | T |
| **18** | **A18** | . | . | . | . | A | . | . | . | . | . | . | . | . | . | . | . | . | . | . | . | . | T | . | . | . | . | . | . | . | . | . | . | . | . | . |
| **19** | **A19** | C | . | A | . | A | T | T | . | . | . | G | . | . | . | G | . | . | . | . | . | . | . | T | G | . | C | C | . | . | . | . | . | A | . | . |
| **20** | **A20** | . | . | . | . | A | T | . | . | . | A | . | . | . | . | . | . | . | . | . | . | . | . | . | . | . | . | . | . | . | . | . | . | . | . | . |
| **21** | **A21** | . | . | . | . | A | T | . | . | . | . | . | . | . | T | . | . | . | . | . | . | . | . | . | . | . | . | . | . | . | . | . | . | . | . | . |
| **22** | **A22** | . | . | . | . | A | T | . | . | . | . | . | . | . | . | . | . | . | . | . | . | . | . | T | . | . | . | C | . | . | C | . | A | . | . | T |
| **23** | **A23** | . | . | . | . | G | T | . | . | . | . | . | . | . | . | . | . | . | . | . | . | . | . | T | . | . | . | C | . | . | C | . | A | . | . | T |
| **24** | **A24** | . | . | . | . | A | T | . | . | . | . | . | . | . | . | . | . | . | . | . | . | . | . | . | . | . | . | . | . | . | . | . | . | . | . | . |
| **25** | **A25** | . | . | . | . | A | T | . | . | . | . | . | . | . | . | . | . | . | . | . | . | . | . | . | G | . | . | . | . | . | . | . | . | . | . | . |
| **26** | **A26** | C | . | A | . | A | T | T | . | . | . | G | . | . | . | G | . | . | . | . | . | . | . | T | G | . | . | C | . | . | . | . | . | . | . | T |
| **27** | **A27** | . | . | . | . | A | . | . | . | . | . | . | . | . | . | . | . | . | . | . | . | . | . | . | . | . | . | . | . | . | . | A | . | . | . | . |
| **28** | **AF197210** | . | . | . | . | G | T | . | . | . | . | . | G | . | . | . | . | . | . | . | . | . | . | T | . | . | C | C | . | . | . | . | . | . | . | . |
| **29** | **AF197211** | . | G | . | . | A | . | . | . | . | . | . | . | . | . | . | . | . | . | . | . | . | . | . | . | . | - | - | . | . | . | . | . | . | . | . |
| **30** | **AF197213** | . | . | . | . | A | T | . | . | . | . | . | G | . | . | . | . | . | . | . | . | . | . | T | . | . | C | C | . | . | . | . | . | . | . | . |
| **31** | **AF197215** | . | . | . | . | A | T | . | . | . | . | . | . | . | . | . | . | . | . | . | . | . | . | . | . | . | . | . | . | . | . | . | . | . | . | . |
| **32** | **AF197216** | . | . | . | . | A | T | . | . | . | . | . | . | . | . | . | . | . | . | . | . | . | . | T | . | . | . | C | . | . | C | . | A | . | . | T |
| **33** | **AF197217** | . | . | . | . | A | T | . | . | . | . | . | . | . | . | . | . | . | . | . | . | . | . | . | . | . | . | . | . | . | . | . | . | . | . | . |
| **34** | **AF475204** | . | . | . | . | A | T | . | . | . | . | . | G | . | . | . | . | . | . | . | . | . | . | T | . | . | . | . | . | . | . | . | . | . | . | . |
| **35** | **AF475206** | . | . | . | . | A | T | . | . | . | . | . | G | . | . | . | . | . | . | . | . | . | . | T | . | . | . | . | . | . | . | . | . | . | . | . |
| **36** | **AF475207** | . | . | . | . | A | T | . | . | . | . | . | . | . | . | . | . | . | . | . | . | . | . | . | . | . | . | . | . | . | . | . | . | . | . | . |
| **37** | **AF475208** | . | . | . | . | A | T | . | . | . | . | . | G | . | . | . | . | . | . | . | . | . | . | T | . | . | . | . | . | . | . | . | . | . | . | . |
| **38** | **AF475209** | . | . | . | . | A | T | . | . | . | . | . | . | . | . | . | . | . | . | . | . | . | . | . | . | . | . | . | . | . | . | . | . | . | . | . |
| **39** | **AF475210** | . | . | . | . | A | T | . | . | . | . | . | . | . | . | . | . | . | . | . | . | . | . | T | . | . | . | C | . | . | C | . | A | . | . | T |
| **40** | **AF475211** | . | . | . | . | A | T | . | . | . | . | G | . | . | . | . | . | . | . | . | . | . | . | T | . | . | . | C | . | . | . | . | . | . | . | . |
| **41** | **AF475212** | . | . | . | . | A | T | . | . | . | . | . | G | . | . | . | . | . | . | . | . | . | . | T | . | . | . | . | . | . | . | . | . | . | . | . |
| **42** | **AF475213** | . | . | . | . | A | T | . | . | . | . | . | . | . | . | . | . | . | . | . | . | . | . | . | . | . | . | . | . | . | . | . | . | . | . | . |
| **43** | **AF475216** | . | . | . | . | A | T | . | . | . | . | . | . | . | . | . | . | . | . | . | . | . | . | . | . | . | . | . | . | . | . | . | . | . | . | . |
| **44** | **AF475218** | . | . | . | . | A | . | . | . | . | . | . | . | . | . | . | . | . | . | . | . | . | . | . | . | . | . | . | . | . | . | . | . | . | . | . |
| **45** | **AF475165** | . | . | . | . | A | T | . | . | . | . | . | . | . | . | . | . | . | . | . | . | . | . | . | . | . | . | . | . | . | . | . | . | . | . | . |
| **46** | **AF475166** | . | . | . | . | A | T | . | . | . | . | . | . | . | . | . | . | . | . | . | . | . | . | T | . | . | . | C | . | . | C | . | A | . | . | T |
| **47** | **AF475169** | . | . | . | . | A | . | . | . | . | . | . | . | . | . | . | . | . | . | . | . | . | . | . | . | . | . | . | . | . | . | . | . | . | . | . |
| **48** | **AF475176** | . | . | . | . | A | T | . | . | . | . | . | . | . | . | . | . | . | . | . | . | . | . | . | . | . | . | . | . | . | . | . | . | . | . | . |
| **49** | **AF475181** | . | . | . | . | A | T | . | . | . | . | . | G | . | . | . | . | . | . | . | . | . | . | T | . | . | . | . | . | . | . | . | . | . | . | . |
| **50** | **AF475185** | . | . | . | . | A | T | . | . | . | . | . | . | . | . | . | . | . | . | . | . | . | . | T | . | . | . | C | . | . | C | . | A | . | . | T |
| **51** | **AF475190** | . | . | . | . | A | T | . | . | . | . | G | . | . | . | . | . | . | . | . | . | . | . | T | . | . | . | C | . | . | . | . | . | . | . | . |
| **52** | **AF475193** | . | . | . | . | A | T | . | . | . | . | . | . | . | . | . | . | . | . | . | . | . | . | . | . | . | . | . | . | . | . | . | . | . | . | . |
| **53** | **AF475195** | . | . | . | . | A | T | . | . | . | . | . | . | . | . | . | . | . | . | . | . | . | . | . | . | . | . | . | . | . | . | . | . | . | . | . |
| **54** | **AF475196** | . | . | . | . | A | T | . | . | . | . | . | G | . | . | . | . | . | . | . | . | . | . | T | . | . | . | . | . | . | . | . | . | . | . | . |
| **55** | **AF475199** | . | . | . | . | A | T | . | . | . | . | G | . | . | . | . | . | . | . | . | . | . | . | T | . | . | . | C | . | . | . | . | . | . | . | . |
| **56** | **AF475223** | . | . | . | . | A | T | . | . | . | . | G | . | . | . | . | . | . | . | . | . | . | . | T | . | . | . | C | . | . | . | . | . | . | . | . |
| **57** | **AF475233** | . | . | . | . | A | T | . | . | . | . | . | . | . | . | . | . | . | . | . | . | . | . | . | . | . | . | . | . | . | . | . | . | . | . | . |
| **58** | **AF475234** | . | . | . | . | A | T | . | . | . | . | . | . | . | . | . | . | . | . | . | . | . | . | T | . | . | . | . | . | . | . | . | . | . | . | . |
| **59** | **AF475235** | . | . | . | . | A | T | . | . | . | . | . | . | . | . | . | . | . | . | . | . | . | . | T | . | . | . | . | . | . | . | . | . | . | . | . |
| **60** | **AF475238** | . | . | . | . | A | . | . | . | . | . | . | . | . | . | . | . | . | . | . | . | . | . | . | . | . | . | . | . | . | . | . | . | . | . | . |
| **61** | **AF475240** | . | . | . | . | A | T | . | . | . | . | G | . | . | . | . | . | . | . | . | . | . | . | T | . | . | . | C | . | . | . | . | . | . | . | . |
| **62** | **AF475241** | . | . | . | . | A | T | . | . | . | . | . | . | . | . | . | . | . | . | . | . | . | . | T | . | . | . | . | . | . | . | . | . | . | . | . |
| **63** | **AF475242** | . | . | . | . | A | T | . | . | . | . | . | . | . | . | . | . | . | . | . | . | . | . | T | . | . | . | . | . | . | . | . | . | . | . | . |
| **64** | **AF475244** | . | . | . | . | A | T | . | . | . | . | G | . | . | . | . | . | . | . | . | . | . | . | T | . | . | . | C | . | . | . | . | . | . | . | . |
| **65** | **AF475248** | . | . | . | . | A | T | . | . | . | . | . | . | . | . | . | . | . | . | . | . | . | . | . | . | . | . | . | . | . | . | . | . | . | . | . |
| **66** | **AF475249** | . | . | . | . | A | . | . | . | . | . | . | . | . | . | . | . | . | . | . | . | . | . | . | . | . | . | . | . | . | . | . | . | . | . | . |
| **67** | **AF475250** | . | . | . | . | A | . | . | . | . | . | . | . | . | . | . | . | . | . | . | . | . | . | . | . | . | . | . | . | . | . | . | . | . | . | . |
| **68** | **AF475251** | . | . | . | . | A | T | . | . | . | . | . | . | . | . | . | . | . | . | . | . | . | . | . | . | . | . | . | . | . | . | . | . | . | . | . |
| **69** | **AF475253** | . | . | . | . | A | . | . | . | . | . | . | . | . | . | . | . | . | . | . | . | . | . | T | . | . | . | C | . | . | C | . | A | . | . | T |
| **70** | **AF475256** | . | . | . | . | A | T | . | . | . | . | . | . | . | . | . | . | . | . | . | . | . | . | T | . | . | . | . | . | . | C | . | A | . | . | T |
| **71** | **AF475257** | . | . | . | . | A | . | . | . | . | . | . | . | . | . | . | . | . | . | . | . | . | . | . | . | . | . | . | . | . | . | . | . | . | . | . |
| **72** | **AF475258** | . | . | . | . | A | T | . | . | . | . | . | . | . | . | . | . | . | . | . | . | . | . | T | . | . | . | C | . | . | . | . | . | . | . | . |
| **73** | **AF475263** | . | . | . | . | A | . | . | . | . | . | . | . | . | . | . | . | . | . | . | . | . | . | . | . | . | . | . | . | . | . | . | . | . | . | . |
| **74** | **AF475264** | . | . | . | . | A | T | . | . | . | . | . | . | . | . | . | . | . | . | . | . | . | . | . | . | . | . | . | . | . | . | . | . | . | . | . |
| **75** | **AF475265** | . | . | . | . | A | T | . | . | . | . | . | G | . | . | . | . | . | . | . | . | . | . | . | . | . | . | . | . | . | . | . | . | . | . | . |
| **76** | **AF475266** | . | . | . | . | A | . | . | . | . | . | . | . | . | . | . | . | . | . | . | . | . | . | . | . | . | . | . | . | . | . | . | . | . | . | . |
| **77** | **AF475278** | . | . | . | . | A | T | . | . | . | . | . | . | . | . | . | . | . | . | . | . | . | . | . | . | . | . | . | . | . | C | . | . | . | . | . |
|  |  |  |  |  |  |  |  |  |  |  |  |  |  |  |  |  |  |  |  |  |  |  |  |  |  |  |  |  |  |  |  |  |  |  |  |  |
| **Hap No:** | **Hap:**  **Name** | **4**  **6**  **1** | **4**  **7**  **5** | **4**  **8**  **4** | **4**  **9**  **3** | **5**  **0**  **7** | **5**  **2**  **2** | **5**  **2**  **8** | **6**  **2**  **0** | **7**  **3**  **4** | **7**  **3**  **6** | **7**  **3**  **8** | **7**  **3**  **9** | **7**  **4**  **6** | **7**  **4**  **8** | **7**  **5**  **0** | **7**  **5**  **5** | **7**  **7**  **7** | **7**  **9**  **8** | **8**  **1**  **3** | **8**  **3**  **2** | **8**  **6**  **7** | **8**  **8**  **3** | **T**  **N**  **O** | **A**  **Z**  **I** | **M**  **U**  **R** | **B**  **H**  **D** | **J**  **A**  **F** | **M**  **E**  **H** | **N**  **A**  **G** | **P**  **A**  **N** | **S**  **U**  **R** | **T**  **O**  **D** |  |  |  |
| **1** | **A01** | T | C | C | A | C | C | G | T | G | G | G | A | G | T | A | T | T | C | C | T | T | C | **8** | **8** | _ | _ | _ | _ | _ | _ | _ | _ |  |  |  |
| **2** | **A02** | . | T | T | G | . | T | A | . | A | . | . | . | . | . | . | . | . | . | . | . | . | . | **5** | **5** | _ | _ | _ | _ | _ | _ | _ | _ |  |  |  |
| **3** | **A03** | . | . | . | . | . | . | . | . | . | . | . | . | . | . | . | . | . | . | . | . | . | . | **1** | **1** | _ | _ | _ | _ | _ | _ | _ | _ |  |  |  |
| **4** | **A04** | . | . | . | . | . | . | A | . | . | . | . | . | . | . | . | . | . | . | . | . | . | . | **15** | **15** | _ | _ | _ | _ | _ | _ | _ | _ |  |  |  |
| **5** | **A05** | C | . | . | . | . | . | . | . | . | . | . | . | . | . | . | . | . | . | . | . | . | . | **1** | **1** | _ | _ | _ | _ | _ | _ | _ | _ |  |  |  |
| **6** | **A06** | . | T | . | . | . | . | . | . | . | . | . | . | . | . | . | . | . | . | . | . | . | . | **1** | **1** | _ | _ | _ | _ | _ | _ | _ | _ |  |  |  |
| **7** | **A07** | . | . | . | . | . | . | A | . | . | T | . | . | . | . | . | . | . | . | . | . | . | . | **1** | **1** | _ | _ | _ | _ | _ | _ | _ | _ |  |  |  |
| **8** | **A08** | . | . | . | . | . | . | A | . | . | . | . | . | . | . | . | . | . | . | . | . | . | . | **3** | **3** | _ | _ | _ | _ | _ | _ | _ | _ |  |  |  |
| **9** | **A09** | . | . | . | . | . | . | . | . | . | . | . | . | . | . | . | . | . | . | . | . | . | . | **1** | **1** | _ | _ | _ | _ | _ | _ | _ | _ |  |  |  |
| **10** | **A10** | . | T | T | G | . | . | A | . | A | . | . | . | . | . | . | . | . | . | . | . | . | . | **1** | **1** | _ | _ | _ | _ | _ | _ | _ | _ |  |  |  |
| **11** | **A11** | . | . | . | . | . | . | . | . | . | . | . | . | . | . | . | . | . | . | . | . | . | . | **2** | **2** | _ | _ | _ | _ | _ | _ | _ | _ |  |  |  |
| **12** | **A12** | . | . | . | . | . | . | . | . | . | . | . | . | . | . | . | . | . | . | . | . | . | . | **2** | **2** | _ | _ | _ | _ | _ | _ | _ | _ |  |  |  |
| **13** | **A13** | . | T | T | G | . | T | A | . | A | . | . | . | . | . | . | . | . | . | . | . | . | . | **2** | **2** | _ | _ | _ | _ | _ | _ | _ | _ |  |  |  |
| **14** | **A14** | . | . | . | . | . | . | . | . | . | . | . | . | . | . | . | . | . | . | . | . | . | . | **1** | **1** | _ | _ | _ | _ | _ | _ | _ | _ |  |  |  |
| **15** | **A15** | . | T | . | . | . | . | A | . | . | . | . | . | A | . | . | . | . | . | . | . | . | . | **5** | **5** | _ | _ | _ | _ | _ | _ | _ | _ |  |  |  |
| **16** | **A16** | . | . | . | . | . | . | . | . | . | . | . | . | . | A | T | . | . | . | . | . | . | . | **2** | **2** | _ | _ | _ | _ | _ | _ | _ | _ |  |  |  |
| **17** | **A17** | . | T | T | G | . | T | A | . | A | . | . | . | . | . | . | . | . | . | . | . | . | . | **1** | **1** | _ | _ | _ | _ | _ | _ | _ | _ |  |  |  |
| **18** | **A18** | . | . | . | . | . | . | . | . | . | . | . | . | . | . | . | . | . | . | . | . | . | . | **1** | **1** | _ | _ | _ | _ | _ | _ | _ | _ |  |  |  |
| **19** | **A19** | . | T | T | G | . | T | A | . | A | . | . | . | . | . | . | . | . | . | . | . | . | . | **3** | **3** | _ | _ | _ | _ | _ | _ | _ | _ |  |  |  |
| **20** | **A20** | . | . | . | . | . | . | A | . | . | . | . | . | . | . | . | . | . | . | . | . | . | . | **1** | **1** | _ | _ | _ | _ | _ | _ | _ | _ |  |  |  |
| **21** | **A21** | . | . | . | . | . | . | A | . | . | . | . | . | . | . | . | . | . | . | . | . | . | . | **2** | **2** | _ | _ | _ | _ | _ | _ | _ | _ |  |  |  |
| **22** | **A22** | . | T | T | G | . | T | A | . | A | . | A | G | . | . | . | . | . | . | . | . | . | . | **1** | **1** | _ | _ | _ | _ | _ | _ | _ | _ |  |  |  |
| **23** | **A23** | . | T | T | G | . | T | A | . | A | . | . | . | . | . | . | . | . | . | . | . | . | . | **1** | **1** | _ | _ | _ | _ | _ | _ | _ | _ |  |  |  |
| **24** | **A24** | . | . | . | . | . | . | . | . | . | . | . | . | . | . | . | . | . | . | . | . | . | . | **1** | **1** | _ | _ | _ | _ | _ | _ | _ | _ |  |  |  |
| **25** | **A25** | . | . | . | . | . | . | A | . | . | . | . | . | . | . | . | . | . | . | . | . | . | . | **1** | **1** | _ | _ | _ | _ | _ | _ | _ | _ |  |  |  |
| **26** | **A26** | . | T | T | G | . | T | A | . | A | . | . | . | . | . | . | . | . | . | . | . | . | . | **2** | **2** | _ | _ | _ | _ | _ | _ | _ | _ |  |  |  |
| **27** | **A27** | . | . | . | . | . | . | . | A | . | . | . | . | . | . | . | . | . | . | . | . | . | . | **1** | **1** | _ | _ | _ | _ | _ | _ | _ | _ |  |  |  |
| **28** | **AF197210** | . | . | . | . | . | . | . | . | . | . | . | . | . | . | . | C | C | . | T | C | C | T | **1** | **_** | 1 | _ | _ | _ | _ | _ | _ | _ |  |  |  |
| **29** | **AF197211** | . | . | . | . | . | . | . | . | . | . | . | . | . | . | . | C | C | . | T | C | C | T | **1** | **_** | 1 | _ | _ | _ | _ | _ | _ | _ |  |  |  |
| **30** | **AF197213** | C | . | . | . | . | . | . | . | . | . | . | . | . | . | . | C | C | . | T | C | C | T | **1** | **_** | 1 | _ | _ | _ | _ | _ | _ | _ |  |  |  |
| **31** | **AF197215** | . | . | T | . | . | . | A | . | . | . | . | . | . | . | . | C | C | A | T | C | C | T | **1** | **_** | 1 | _ | _ | _ | _ | _ | _ | _ |  |  |  |
| **32** | **AF197216** | . | T | T | G | . | T | A | . | A | . | . | . | . | . | . | C | C | . | T | . | C | T | **6** | **_** | 1 | _ | _ | 2 | _ | _ | _ | 3 |  |  |  |
| **33** | **AF197217** | . | . | . | . | . | . | A | . | . | . | . | . | . | . | . | C | C | A | T | C | C | T | **1** | **_** | 1 | _ | _ | _ | _ | _ | _ | _ |  |  |  |
| **34** | **AF475204** | . | . | . | . | . | . | . | . | . | . | . | . | . | . | . | C | C | . | T | C | C | T | **3** | **_** | 3 | _ | _ | _ | _ | _ | _ | _ |  |  |  |
| **35** | **AF475206** | . | T | . | . | . | T | A | . | A | . | . | . | . | . | . | C | C | . | T | . | C | T | **2** | **_** | 2 | _ | _ | _ | _ | _ | _ | _ |  |  |  |
| **36** | **AF475207** | . | . | . | . | . | T | A | . | . | . | . | . | . | . | . | C | C | . | T | C | C | T | **1** | **_** | 1 | _ | _ | _ | _ | _ | _ | _ |  |  |  |
| **37** | **AF475208** | . | T | . | . | . | T | A | . | . | . | . | . | . | . | . | C | C | . | T | C | C | T | **1** | **_** | 1 | _ | _ | _ | _ | _ | _ | _ |  |  |  |
| **38** | **AF475209** | . | . | . | . | . | . | A | . | . | . | . | . | . | . | . | C | C | . | T | C | C | T | **10** | **_** | 2 | 3 | 1 | _ | 2 | 2 | _ | _ |  |  |  |
| **39** | **AF475210** | . | T | T | G | . | . | . | . | . | . | . | . | . | . | . | C | C | . | T | C | C | T | **6** | **_** | 1 | _ | 2 | 1 | 2 | _ | _ | _ |  |  |  |
| **40** | **AF475211** | . | T | T | G | T | . | A | . | . | . | . | . | . | . | . | C | C | . | T | C | C | T | **1** | **_** | 1 | _ | _ | _ | _ | _ | _ | _ |  |  |  |
| **41** | **AF475212** | . | T | . | . | . | . | A | . | . | . | . | . | . | . | . | C | C | . | T | C | C | T | **1** | **_** | _ | _ | _ | _ | _ | _ | _ | _ |  |  |  |
| **42** | **AF475213** | . | . | . | . | . | . | . | . | . | . | . | . | . | . | . | C | C | . | T | C | C | T | **15** | **_** | 1 | 5 | 3 | _ | 4 | 1 | 1 | _ |  |  |  |
| **43** | **AF475216** | . | . | . | . | T | . | A | . | . | . | . | . | . | . | . | C | C | . | T | C | C | T | **3** | **_** | 1 | _ | _ | 2 | _ | _ | _ | _ |  |  |  |
| **44** | **AF475218** | . | . | . | . | . | . | . | . | . | . | . | . | . | . | . | C | C | . | T | C | C | T | **7** | **_** | 1 | 2 | _ | _ | _ | _2 | 2 | _ |  |  |  |
| **45** | **AF475165** | . | T | . | . | . | . | . | . | . | . | . | . | . | . | . | C | C | . | T | C | C | T | **4** | **_** | _ | 1 | 2 | 1 | _ | _ | _ | _ |  |  |  |
| **46** | **AF475166** | . | T | T | G | . | T | A | . | A | . | . | . | . | . | . | C | C | . | T | . | C | T | **8** | **_** | _ | 3 | 4 | _ | 1 | _ | _ | _ |  |  |  |
| **47** | **AF475169** | . | . | . | . | T | . | . | . | . | . | . | . | . | . | . | C | C | . | T | C | C | T | **2** | **_** | _ | 1 | _ | 1 | _ | _ | _ | _ |  |  |  |
| **48** | **AF475176** | . | . | . | . | . | T | A | . | A | . | . | . | . | . | . | C | C | . | T | . | C | T | **4** | **_** | _ | _ | 1 | _ | 2 | _ | 1 | _ |  |  |  |
| **49** | **AF475181** | . | T | . | . | . | . | . | . | . | . | . | . | . | . | . | C | C | . | T | C | C | T | **3** | **_** | _ | _ | 1 | _ | 2 | _ | _ | _ |  |  |  |
| **50** | **AF475185** | . | T | T | G | . | . | A | . | . | . | . | . | . | . | . | C | C | . | T | C | C | T | **4** | **_** | _ | _ | 1 | 2 | _ | 1 | _ | _ |  |  |  |
| **51** | **AF475190** | . | T | T | G | T | . | . | . | . | . | . | . | . | . | . | C | C | . | T | C | C | T | **2** | **_** | _ | _ | _ | 2 | _ | _ | _ | _ |  |  |  |
| **52** | **AF475193** | . | . | T | . | T | . | A | . | . | . | . | . | . | . | . | C | C | . | T | C | C | T | **1** | **_** | _ | _ | _ | 1 | _ | _ | _ | _ |  |  |  |
| **53** | **AF475195** | . | . | . | . | T | . | . | . | . | . | . | . | . | . | . | C | C | . | T | C | C | T | **1** | **_** | _ | _ | _ | 1 | _ | _ | _ | _ |  |  |  |
| **54** | **AF475196** | . | . | . | . | . | T | A | . | A | . | . | . | . | . | . | C | C | . | T | . | C | T | **1** | **_** | _ | _ | _ | 1 | _ | _ | _ | _ |  |  |  |
| **55** | **AF475199** | . | T | T | G | . | . | A | . | . | . | . | . | . | . | . | C | C | . | T | C | C | T | **2** | **_** | _ | _ | _ | 1 | _ | _ | 1 | _ |  |  |  |
| **56** | **AF475223** | . | T | T | G | . | . | . | . | . | . | . | . | . | . | . | C | C | . | T | C | C | T | **1** | **_** | _ | _ | _ | _ | 1 | _ | _ | _ |  |  |  |
| **57** | **AF475233** | . | T | . | . | . | . | A | . | . | . | . | . | . | . | . | C | C | . | T | C | C | T | **1** | **_** | _ | _ | _ | _ | 1 | _ | _ | _ |  |  |  |
| **58** | **AF475234** | . | . | . | . | . | T | A | . | A | . | . | . | . | . | . | C | C | . | T | . | C | T | **2** | **_** | _ | _ | _ | _ | 2 | _ | _ | _ |  |  |  |
| **59** | **AF475235** | . | . | . | . | T | . | A | . | . | . | . | . | . | . | . | C | C | . | T | C | C | T | **1** | **_** | _ | _ | _ | _ | 1 | _ | _ | _ |  |  |  |
| **60** | **AF475238** | . | . | . | . | T | T | A | . | . | . | . | . | . | . | . | C | C | . | T | C | C | T | **1** | **_** | _ | _ | _ | _ | 1 | _ | _ | _ |  |  |  |
| **61** | **AF475240** | . | T | T | G | . | T | A | . | A | . | . | . | . | . | . | C | C | . | T | . | C | T | **2** | **_** | _ | _ | _ | _ | 1 | _ | _ | 1 |  |  |  |
| **62** | **AF475241** | . | . | . | . | . | . | A | . | . | . | . | . | . | . | . | C | C | . | T | C | C | T | **1** | **_** | _ | _ | _ | _ | 1 | _ | _ | _ |  |  |  |
| **63** | **AF475242** | . | . | . | . | . | . | . | . | . | . | . | . | . | . | . | C | C | . | T | C | C | T | **1** | **_** | _ | _ | _ | _ | 1 | _ | _ | _ |  |  |  |
| **64** | **AF475244** | . | T | T | G | . | . | . | . | . | . | . | . | . | . | . | C | C | . | T | C | C | T | **1** | **_** | _ | _ | _ | _ | 1 | _ | _ | _ |  |  |  |
| **65** | **AF475248** | . | T | . | . | . | T | A | . | . | . | . | . | . | . | . | C | C | . | T | . | C | T | **1** | **_** | _ | _ | _ | _ | 1 | _ | _ | _ |  |  |  |
| **66** | **AF475249** | . | . | . | . | T | . | A | . | . | . | . | . | . | . | . | C | C | . | T | C | C | T | **1** | **_** | _ | _ | _ | _ | _ | 1 | _ | _ |  |  |  |
| **67** | **AF475250** | . | T | . | . | . | . | . | . | . | . | . | . | . | . | . | C | C | . | T | C | C | T | **2** | **_** | _ | _ | _ | _ | _ | 2 | _ | _ |  |  |  |
| **68** | **AF475251** | . | T | . | . | . | T | . | . | . | . | . | . | . | . | . | C | C | . | T | C | C | T | **1** | **_** | _ | _ | _ | _ | _ | 1 | _ | _ |  |  |  |
| **69** | **AF475253** | . | T | T | G | . | T | A | . | A | . | . | . | . | . | . | C | C | . | T | . | C | T | **1** | **_** | _ | _ | _ | _ | _ | 1 | _ | _ |  |  |  |
| **70** | **AF475256** | . | T | T | G | . | . | A | . | . | . | . | . | . | . | . | C | C | . | T | C | C | T | **2** | **_** | _ | _ | _ | _ | _ | 2 | _ | _ |  |  |  |
| **71** | **AF475257** | . | T | . | G | . | . | . | . | . | . | . | . | . | . | . | C | C | . | T | C | C | T | **1** | **_** | _ | _ | _ | _ | _ | 1 | _ | _ |  |  |  |
| **72** | **AF475258** | . | T | T | G | . | T | . | . | A | . | . | . | . | . | . | C | C | . | T | . | C | T | **1** | **_** | _ | _ | _ | _ | _ | 1 | _ | _ |  |  |  |
| **73** | **AF475263** | . | . | . | . | . | T | . | . | . | . | . | . | . | . | . | C | C | . | T | C | C | T | **1** | **_** | _ | _ | _ | _ | _ | 1 | _ | _ |  |  |  |
| **74** | **AF475264** | . | . | . | . | . | . | A | . | . | . | . | . | . | . | . | C | C | . | T | C | C | T | **4** | **_** | _ | _ | _ | _ | _ | _ | 4 | _ |  |  |  |
| **75** | **AF475265** | . | T | . | . | . | . | A | . | . | . | . | . | . | . | . | C | C | . | T | C | C | T | **4** | **_** | _ | _ | _ | _ | _ | _ | 4 | _ |  |  |  |
| **76** | **AF475266** | . | . | . | . | . | . | . | . | . | . | . | . | . | . | . | C | C | . | T | C | C | T | **2** | **_** | _ | _ | _ | _ | _ | _ | 2 | _ |  |  |  |
| **77** | **AF475278** | . | T | . | . | . | . | A | . | . | . | . | . | . | . | . | C | C | . | T | C | C | T | **1** | **_** | _ | _ | _ | _ | _ | _ | 1 | _ |  |  |  |

Abbreviations: Hap: (Haplotypes) T. No: (Total Number of Haplotypes) AZI: (Azikheli) MUR: (MURRAH) BHD: (BHADHAWARI)

JAF: (JAFFARABADI) MEH: (MEHSANA) NAG: (NAGPURI) PAN: (PANDHARPURI) SUR: (SURTI) TOD: (TODA)
