# Supplementary material for: Genetic Diversity Relationship in Azakheli Buffalo Inferred from mtDNA and MC1R Sequences Comparison
Source: Biomed Res Int. 2022 Dec 13;2022:5770562. doi: 10.1155/2022/5770562 (PMC9806686; doi:10.1155/2022/5770562)
Supplement: Supplementary 4 — Supplementary Table S4: data on 68 sequences of Azakheli buffalo of MC1R haplotypes including 198 Indian buffaloes and 142 Chinese buffalo sequences. The phenotype of all individuals was defined in the table. Chinese and Indian buffaloes MC1R haplotype sequences retrieved from GenBank were used for the median-joining network analysis (Figure 3). [file 5770562.f4.docx]

**Supplementary Table S4.** Data on 68 sequences of Azakheli buffalo of *MC1R* haplotypes including 198 Indian Buffaloes and 142 Chinese buffalo sequences. The coat color of all individuals was defined in the table. Chinese and Indian Buffaloes *MC1R* haplotype sequences retrieved from GenBank were used for the median joining network analysis (Figure. 3).

| **S/No** | **Sequence Names/**  **Accession No** | **Breed** | **Color** | **Hap: No** | **Location** |
| --- | --- | --- | --- | --- | --- |
| 1 | Mc1r-169 | Azikheli | Piebald | H1 | Pakistan |
| 2 | Mc1r-168 | Azikheli | Piebald | H1 | Pakistan |
| 3 | Mc1r-166 | Azikheli | Piebald | H1 | Pakistan |
| 4 | Mc1r-137 | Azikheli | Piebald | H1 | Pakistan |
| 5 | Mc1r-118 | Azikheli | Brown And White | H2 | Pakistan |
| 6 | Mc1r-356 | Azikheli | Gray And White | H3 | Pakistan |
| 7 | Mc1r-351 | Azikheli | Gray | H4 | Pakistan |
| 8 | Mc1r-331 | Azikheli | Albino | H5 | Pakistan |
| 9 | Mc1r-330 | Azikheli | Gray And White | H3 | Pakistan |
| 10 | Mc1r-329 | Azikheli | Piebald | H6 | Pakistan |
| 11 | Mc1r-315 | Azikheli | Gray And White | H3 | Pakistan |
| 12 | Mc1r-313 | Azikheli | Gray And White | H3 | Pakistan |
| 13 | Mc1r-311 | Azikheli | Black | H7 | Pakistan |
| 14 | Mc1r-310 | Azikheli | Brown | H8 | Pakistan |
| 15 | Mc1r-309 | Azikheli | Brown And White | H9 | Pakistan |
| 16 | Mc1r-308 | Azikheli | Black | H7 | Pakistan |
| 17 | Mc1r-307 | Azikheli | Gray And White | H10 | Pakistan |
| 18 | Mc1r-306 | Azikheli | Black | H9 | Pakistan |
| 19 | Mc1r-305 | Azikheli | Black | H9 | Pakistan |
| 20 | Mc1r-304 | Azikheli | Black | H9 | Pakistan |
| 21 | Mc1r-303 | Azikheli | Black | H9 | Pakistan |
| 22 | Mc1r-302 | Azikheli | Black | H9 | Pakistan |
| 23 | Mc1r-301 | Azikheli | Black | H9 | Pakistan |
| 24 | Mc1r-300 | Azikheli | Black | H9 | Pakistan |
| 25 | Mc1r-298 | Azikheli | Albino | H11 | Pakistan |
| 26 | Mc1r-296 | Azikheli | Piebald | H1 | Pakistan |
| 27 | Mc1r-S9 | Azikheli | Piebald | H1 | Pakistan |
| 28 | Mc1r-S7 | Azikheli | Albino | H12 | Pakistan |
| 29 | Mc1r-S6 | Azikheli | Gray And White | H3 | Pakistan |
| 30 | Mc1r-117 | Azikheli | Piebald | H6 | Pakistan |
| 31 | Mc1r-116 | Azikheli | Black | H9 | Pakistan |
| 32 | Mc1r-115 | Azikheli | Black | H9 | Pakistan |
| 33 | Mc1r-114 | Azikheli | Black | H9 | Pakistan |
| 34 | Mc1r-113 | Azikheli | Black | H9 | Pakistan |
| 35 | Mc1r-112 | Azikheli | Black | H9 | Pakistan |
| 36 | Mc1r-111 | Azikheli | Black | H9 | Pakistan |
| 37 | Mc1r-110 | Azikheli | Black | H9 | Pakistan |
| 38 | Mc1r-106 | Azikheli | Brown And White | H14 | Pakistan |
| 39 | Mc1r-105 | Azikheli | Gray And White | H15 | Pakistan |
| 40 | Mc1r-104 | Azikheli | Black | H9 | Pakistan |
| 41 | Mc1r-103 | Azikheli | Black | H9 | Pakistan |
| 42 | Mc1r-102 | Azikheli | Brown | H16 | Pakistan |
| 43 | Mc1r-101 | Azikheli | Black | H9 | Pakistan |
| 44 | Mc1r-100 | Azikheli | Black | H9 | Pakistan |
| 45 | Mc1r-99 | Azikheli | Black | H9 | Pakistan |
| 46 | Mc1r-97 | Azikheli | Black | H9 | Pakistan |
| 47 | Mc1r-78 | Azikheli | Black | H9 | Pakistan |
| 48 | Mc1r-88 | Azikheli | Black | H9 | Pakistan |
| 49 | Mc1r-87 | Azikheli | Black | H9 | Pakistan |
| 50 | Mc1r-01 | Azikheli | Black | H9 | Pakistan |
| 51 | Mc1r-25 | Azikheli | Black | H9 | Pakistan |
| 52 | Mc1r-23 | Azikheli | Black | H9 | Pakistan |
| 53 | Mc1r-02 | Azikheli | Black | H9 | Pakistan |
| 54 | Mc1r-31 | Azikheli | Gray And White | H17 | Pakistan |
| 55 | Mc1r-35 | Azikheli | Gray And White | H3 | Pakistan |
| 56 | Mc1r-38 | Azikheli | Black | H9 | Pakistan |
| 57 | Mc1r-41 | Azikheli | Brown | H18 | Pakistan |
| 58 | Mc1r-44 | Azikheli | Brown | H18 | Pakistan |
| 59 | Mc1r-48 | Azikheli | Brown And White | H19 | Pakistan |
| 60 | Mc1r-45 | Azikheli | Black | H9 | Pakistan |
| 61 | Mc1r-52 | Azikheli | Brown And White | H19 | Pakistan |
| 62 | Mc1r-53 | Azikheli | Brown And White | H19 | Pakistan |
| 63 | Mc1r-61 | Azikheli | Brown | H20 | Pakistan |
| 64 | Mc1r-69 | Azikheli | Brown And White | H19 | Pakistan |
| 65 | Mc1r-71 | Azikheli | Brown And White | H19 | Pakistan |
| 66 | Mc1r-73 | Azikheli | Black | H9 | Pakistan |
| 67 | Mc1r-77 | Azikheli | Black | H9 | Pakistan |
| 68 | Mc1r-75 | Azikheli | Brown | H16 | Pakistan |
| 69 | Gq359864.1 2007003 | Chinese Swamp Buffalo | White | H21 | China |
| 70 | Gq359865.1 2007031 | Chinese Swamp Buffalo | White | H21 | China |
| 71 | Gq359866.1 Dn15 | Chinese Swamp Buffalo | White | H21 | China |
| 72 | Gq359867.1 2007018 | Chinese Swamp Buffalo | White | H21 | China |
| 73 | Gq359869.1 2007020 | Chinese Swamp Buffalo | White | H21 | China |
| 74 | Gq359870 | Chinese Swamp Buffalo | White | H10 | China |
| 75 | Gq359871.1 2007001 | Chinese Swamp Buffalo | White | H21 | China |
| 76 | Gq359873.1 2007017 | Chinese Swamp Buffalo | White | H21 | China |
| 77 | Gq359874.1 Dn12 | Chinese Swamp Buffalo | White | H21 | China |
| 78 | Gq359876.1 Dn01 | Chinese Swamp Buffalo | White | H21 | China |
| 79 | Gq359878.1 2007016 | Chinese Swamp Buffalo | White | H21 | China |
| 80 | Gq359879 | Chinese Swamp Buffalo | Gray | H10 | China |
| 81 | Gq359880.1 2007013 | Chinese Swamp Buffalo | White | H21 | China |
| 82 | Gu121238.1 Dc18 | Chinese Swamp Buffalo | Gray | H21 | China |
| 83 | Gu121239.1 Dc27 | Chinese Swamp Buffalo | Gray | H21 | China |
| 84 | Gu121240.1 Dc4 | Chinese Swamp Buffalo | Gray | H21 | China |
| 85 | Gu121241.1 Dc9 | Chinese Swamp Buffalo | Gray | H21 | China |
| 86 | Gu121242.1 Ddn11 | Chinese Swamp Buffalo | Gray | H21 | China |
| 87 | Gu121243.1 Ddn37 | Chinese Swamp Buffalo | Gray | H21 | China |
| 88 | Gu121244.1 Ddn39 | Chinese Swamp Buffalo | Gray | H21 | China |
| 89 | Gu121245.1 Ddn40 | Chinese Swamp Buffalo | Gray | H21 | China |
| 90 | Gu121246.1 Ddn43 | Chinese Swamp Buffalo | Gray | H21 | China |
| 91 | Gu121247.1 Ddn44 | Chinese Swamp Buffalo | Gray | H21 | China |
| 92 | Gu121248.1 Ddn50 | Chinese Swamp Buffalo | Gray | H21 | China |
| 93 | Gu121249.1 Ddn13 | Chinese Swamp Buffalo | Gray | H21 | China |
| 94 | Gu121250.1 Ddn24 | Chinese Swamp Buffalo | Gray | H21 | China |
| 95 | Gu121251.1 G7 | Chinese Swamp Buffalo | Gray | H21 | China |
| 96 | Gu121252.1 Gz1 | Chinese Swamp Buffalo | Gray | H21 | China |
| 97 | Gu121253.1 Gz15 | Chinese Swamp Buffalo | Gray | H21 | China |
| 98 | Gu121254.1 G12 | Chinese Swamp Buffalo | Gray | H21 | China |
| 99 | Gu121255.1 Gz13 | Chinese Swamp Buffalo | Gray | H21 | China |
| 100 | Gu121256.1 Ddn45 | Chinese Swamp Buffalo | Gray | H21 | China |
| 101 | Gu121259.1 Ddn42 | Chinese Swamp Buffalo | Gray | H21 | China |
| 102 | Gu121258.1 Ddn4 | Chinese Swamp Buffalo | Gray | H21 | China |
| 103 | Gu121257.1 Ddn9 | Chinese Swamp Buffalo | Gray | H21 | China |
| 104 | Gu121260.1 Ddn41 | Chinese Swamp Buffalo | Gray | H21 | China |
| 105 | Gu121261.1 Ddn46 | Chinese Swamp Buffalo | Gray | H21 | China |
| 106 | Gu121262.1 Ddn30 | Chinese Swamp Buffalo | Gray | H21 | China |
| 107 | Gu121263.1 Ddn32 | Chinese Swamp Buffalo | Gray | H21 | China |
| 108 | Gu121264.1 Ddn25 | Chinese Swamp Buffalo | Gray | H21 | China |
| 109 | Gu121265.1 Ddn29 | Chinese Swamp Buffalo | Gray | H21 | China |
| 110 | Gu121267.1 Ddn33 | Chinese Swamp Buffalo | Gray | H21 | China |
| 111 | Gu121266.1 Ddn14 | Chinese Swamp Buffalo | Gray | H21 | China |
| 112 | Gu121268.1 Dc52 | Chinese Swamp Buffalo | Gray | H21 | China |
| 113 | Gu121269.1 Dc7 | Chinese Swamp Buffalo | Gray | H21 | China |
| 114 | Gu121270.1 Dc46 | Chinese Swamp Buffalo | Gray | H21 | China |
| 115 | Gu121271.1 Dc51 | Chinese Swamp Buffalo | Gray | H21 | China |
| 116 | Gu121272.1 Dc40 | Chinese Swamp Buffalo | Gray | H21 | China |
| 117 | Gu121273.1 Dc43 | Chinese Swamp Buffalo | Gray | H21 | China |
| 118 | Gu121274.1 Dc20 | Chinese Swamp Buffalo | Gray | H21 | China |
| 119 | Gu121275.1 Dc28 | Chinese Swamp Buffalo | Gray | H21 | China |
| 120 | Gu121276.1 Dc25 | Chinese Swamp Buffalo | Gray | H21 | China |
| 121 | Gu121277.1 Gz9 | Chinese Swamp Buffalo | Gray | H21 | China |
| 122 | Gu121278.1 Gz34 | Chinese Swamp Buffalo | Gray | H21 | China |
| 123 | Gu121279.1 G22 | Chinese Swamp Buffalo | Gray | H21 | China |
| 124 | Gu121280.1 Gz10 | Chinese Swamp Buffalo | Gray | H21 | China |
| 125 | Gu121281.1 G2 | Chinese Swamp Buffalo | Gray | H21 | China |
| 126 | Gu121282.1 G27 | Chinese Swamp Buffalo | Gray | H21 | China |
| 127 | Gu121283.1 G31 | Chinese Swamp Buffalo | Gray | H21 | China |
| 128 | Gu121284.1 Gz14 | Chinese Swamp Buffalo | Gray | H21 | China |
| 129 | Gu121285.1 G17 | Chinese Swamp Buffalo | Gray | H21 | China |
| 130 | Gu121286.1 Gz36 | Chinese Swamp Buffalo | Gray | H21 | China |
| 131 | Gu121287.1 G8 | Chinese Swamp Buffalo | Gray | H21 | China |
| 132 | Gu121288.1 Gz2 | Chinese Swamp Buffalo | Gray | H21 | China |
| 133 | Gu121289.1 G36 | Chinese Swamp Buffalo | Gray | H21 | China |
| 134 | Gu121290.1 G26 | Chinese Swamp Buffalo | Gray | H21 | China |
| 135 | Gu121291.1 Gz8 | Chinese Swamp Buffalo | Gray | H21 | China |
| 136 | Gu121292.1 G28 | Chinese Swamp Buffalo | Gray | H21 | China |
| 137 | Gu121293.1 G16 | Chinese Swamp Buffalo | Gray | H21 | China |
| 138 | Gu121294.1 Gz7 | Chinese Swamp Buffalo | Gray | H21 | China |
| 139 | Gu121295.1 Gz3 | Chinese Swamp Buffalo | Gray | H21 | China |
| 140 | Gu121296.1 Gz11 | Chinese Swamp Buffalo | Gray | H21 | China |
| 141 | Gu121297.1 Gz12 | Chinese Swamp Buffalo | Gray | H21 | China |
| 142 | Gu121298.1 G20 | Chinese Swamp Buffalo | Gray | H21 | China |
| 143 | Gu121299.1 G18 | Chinese Swamp Buffalo | Gray | H21 | China |
| 144 | Gu121300.1 Gz6 | Chinese Swamp Buffalo | Gray | H21 | China |
| 145 | Gu121301.1 G15 | Chinese Swamp Buffalo | Gray | H21 | China |
| 146 | Gq359872.1 2007047 | Chinese Swamp Buffalo | Gray | H21 | China |
| 147 | Gq359875.1 2007059 | Chinese River Buffalo | Black | H9 | China |
| 148 | Gq359887.1 2007060 | Chinese River Buffalo | Black | H9 | China |
| 149 | Gu121327.1 Ml1 | Chinese River Buffalo | Black | H9 | China |
| 150 | Gu121328.1 Ml2 | Chinese River Buffalo | Black | H9 | China |
| 151 | Gu121329.1 Ml3 | Chinese River Buffalo | Black | H9 | China |
| 152 | Gu121330.1 Ml4 | Chinese River Buffalo | Black | H9 | China |
| 153 | Gu121331.1 Ml5 | Chinese River Buffalo | Black | H9 | China |
| 154 | Gu121332.1 Ml6 | Chinese River Buffalo | Black | H9 | China |
| 155 | Gu121333.1 Ml7 | Chinese River Buffalo | Black | H9 | China |
| 156 | Gu121334.1 07-50 | Chinese River Buffalo | Black | H9 | China |
| 157 | Gu121335.1 Ml9 | Chinese River Buffalo | Black | H9 | China |
| 158 | Gu121336.1 07-44 | Chinese River Buffalo | Black | H9 | China |
| 159 | Gu121337.1 07-54 | Chinese River Buffalo | Black | H9 | China |
| 160 | Gu121338.1 07-61 | Chinese River Buffalo | Black | H9 | China |
| 161 | Gu121339.1 Ml13 | Chinese River Buffalo | Black | H9 | China |
| 162 | Gu121340.1 07-56 | Chinese River Buffalo | Black | H9 | China |
| 163 | Gu121341.1 Ml14 | Chinese River Buffalo | Black | H9 | China |
| 164 | Gu121342.1 Nl19 | Chinese River Buffalo | Black | H9 | China |
| 165 | Gu121343.1 Nl3 | Chinese River Buffalo | Black | H9 | China |
| 166 | Gu121344.1 Nl6 | Chinese River Buffalo | Black | H9 | China |
| 167 | Gu121345.1 Ml17 | Chinese River Buffalo | Black | H9 | China |
| 168 | Gu121346.1 07-49 | Chinese River Buffalo | Black | H9 | China |
| 169 | Gu121347.1 Ml8 | Chinese River Buffalo | Black | H9 | China |
| 170 | Gu121348.1 07-55 | Chinese River Buffalo | Black | H9 | China |
| 171 | Gu121349.1 07-42 | Chinese River Buffalo | Black | H9 | China |
| 172 | Gu121350.1 Ml10 | Chinese River Buffalo | Black | H9 | China |
| 173 | Gu121351.1 07-45 | Chinese River Buffalo | Black | H9 | China |
| 174 | Gu121352.1 Ml11 | Chinese River Buffalo | Black | H9 | China |
| 175 | Gu121353.1 Nl4 | Chinese River Buffalo | Black | H9 | China |
| 176 | Gu121354.1 Nl7 | Chinese River Buffalo | Black | H9 | China |
| 177 | Gu121355.1 Ml18 | Chinese River Buffalo | Black | H9 | China |
| 178 | Gu121356.1 Ml20 | Chinese River Buffalo | Black | H9 | China |
| 179 | Gu121357.1 Ml12 | Chinese River Buffalo | Black | H9 | China |
| 180 | Gu121358.1 Nl5 | Chinese River Buffalo | Black | H9 | China |
| 181 | Gu121359.1 Nl2 | Chinese River Buffalo | Black | H9 | China |
| 182 | Gu121360.1 Nl16 | Chinese River Buffalo | Black | H9 | China |
| 183 | Gu121361.1 Nl15 | Chinese River Buffalo | Black | H9 | China |
| 184 | Gu121362.1 Nl1 | Chinese River Buffalo | Black | H9 | China |
| 185 | Kp031113.1 Nicra 11 | Indian River Buffalo | Black | H9 | India |
| 186 | Kp182075.1 Nicra-27 | Indian River Buffalo | Black | H9 | India |
| 187 | Kp182076.1 Nicra-28 | Indian River Buffalo | Black | H9 | India |
| 188 | Kp182077.1 Nicra-29 | Indian River Buffalo | Black | H9 | India |
| 189 | Kp182078.1 Nicra-30 | Indian River Buffalo | Black | H9 | India |
| 190 | Kp182079.1 Nicra-31 | Indian River Buffalo | Black | H9 | India |
| 191 | Kp347596.1 Nicra-49 | Indian River Buffalo | Black | H9 | India |
| 192 | Kp347597.1 Nicra-50 | Indian River Buffalo | Black | H9 | India |
| 193 | Kp347598.1 Nicra-51 | Indian River Buffalo | Black | H9 | India |
| 194 | Kp347599.1 Nicra-52 | Indian River Buffalo | Black | H9 | India |
| 195 | Kp347600.1 Nicra-53 | Indian River Buffalo | Black | H9 | India |
| 196 | Kp347601.1 Nicra-54 | Indian River Buffalo | Black | H9 | India |
| 197 | Kp347602.1 Nicra-55 | Indian River Buffalo | Black | H9 | India |
| 198 | Kp347603.1 Nicra-56 | Indian River Buffalo | Black | H9 | India |
| 199 | Mf421323.1 Nicra-308 | Indian River Buffalo | Black | H9 | India |
| 200 | Mf421324.1 Nicra-309 | Indian River Buffalo | Black | H9 | India |
| 201 | Mf421325.1 Nicra-310 | Indian River Buffalo | Black | H9 | India |
| 202 | Mf421327.1 Nicra-312 | Indian River Buffalo | Black | H9 | India |
| 203 | Mf421326.1 Nicra-311 | Indian River Buffalo | Black | H9 | India |
| 204 | Mf421328.1 Nicra-313 | Indian River Buffalo | Black | H9 | India |
| 205 | Mf421329.1 Nicra-314 | Indian River Buffalo | Black | H9 | India |
| 206 | Mf421330.1 Nicra-315 | Indian River Buffalo | Black | H9 | India |
| 207 | Mf421331.1 Nicra-316 | Indian River Buffalo | Black | H9 | India |
| 208 | Mf421332.1 Nicra-317 | Indian River Buffalo | Black | H9 | India |
| 209 | Mf421333.1 Nicra-318 | Indian River Buffalo | Black | H9 | India |
| 210 | Mf421334.1 Nicra-319 | Indian River Buffalo | Black | H9 | India |
| 211 | Mf421335.1 Nicra-320 | Indian River Buffalo | Black | H9 | India |
| 212 | Mf421336.1 Nicra-321 | Indian River Buffalo | Black | H9 | India |
| 213 | Mf421337.1 Nicra-322 | Indian River Buffalo | Black | H9 | India |
| 214 | Mf421338.1 Nicra-323 | Indian River Buffalo | Black | H9 | India |
| 215 | Mf421339.1 Nicra-324 | Indian River Buffalo | Black | H9 | India |
| 216 | Mf421340.1 Nicra-325 | Indian River Buffalo | Black | H9 | India |
| 217 | Mf421341.1 Nicra-326 | Indian River Buffalo | Black | H9 | India |
| 218 | Mf421342.1 Nicra-327 | Indian River Buffalo | Black | H9 | India |
| 219 | Mf421343.1 Nicra-328 | Indian River Buffalo | Black | H9 | India |
| 220 | Mf421344.1 Nicra-329 | Indian River Buffalo | Black | H9 | India |
| 221 | Mf421345.1 Nicra-330 | Indian River Buffalo | Black | H9 | India |
| 222 | Mf421346.1 Nicra-331 | Indian River Buffalo | Black | H9 | India |
| 223 | Mf421347.1 Nicra-332 | Indian River Buffalo | Black | H9 | India |
| 224 | Mf421348.1 Nicra-333 | Indian River Buffalo | Black | H9 | India |
| 225 | Mf421349.1 Nicra-334 | Indian River Buffalo | Black | H9 | India |
| 226 | Mf421350.1 Nicra-335 | Indian River Buffalo | Black | H9 | India |
| 227 | Mf421351.1 Nicra-336 | Indian River Buffalo | Black | H9 | India |
| 228 | Mf421352.1 Nicra-337 | Indian River Buffalo | Black | H9 | India |
| 229 | Mf421353.1 Nicra-338 | Indian River Buffalo | Black | H9 | India |
| 230 | Mf421354.1 Nicra-339 | Indian River Buffalo | Black | H9 | India |
| 231 | Mf421355.1 Nicra-340 | Indian River Buffalo | Black | H9 | India |
| 232 | Mf421356.1 Nicra-341 | Indian River Buffalo | Black | H9 | India |
| 233 | Mf421357.1 Nicra-342 | Indian River Buffalo | Black | H9 | India |
| 234 | Mf421358.1 Nicra-343 | Indian River Buffalo | Black | H9 | India |
| 235 | Mf421359.1 Nicra-344 | Indian River Buffalo | Black | H9 | India |
| 236 | Mf421360.1 Nicra-345 | Indian River Buffalo | Black | H9 | India |
| 237 | Mf421361.1 Nicra-346 | Indian River Buffalo | Black | H9 | India |
| 238 | Mf421362.1 Nicra-347 | Indian River Buffalo | Black | H9 | India |
| 239 | Mf421363.1 Nicra-348 | Indian River Buffalo | Black | H9 | India |
| 240 | Mf421364.1 Nicra-349 | Indian River Buffalo | Black | H9 | India |
| 241 | Mf421365.1 Nicra-350 | Indian River Buffalo | Black | H9 | India |
| 242 | Mf421366.1 Nicra-351 | Indian River Buffalo | Black | H9 | India |
| 243 | Mf421367.1 Nicra-352 | Indian River Buffalo | Black | H9 | India |
| 244 | Mf421368.1 Nicra-353 | Indian River Buffalo | Black | H9 | India |
| 245 | Mf421369.1 Nicra-354 | Indian River Buffalo | Black | H9 | India |
| 246 | Mf421370.1 Nicra-355 | Indian River Buffalo | Black | H9 | India |
| 247 | Mf421371.1 Nicra-356 | Indian River Buffalo | Black | H9 | India |
| 248 | Mf421372.1 Nicra-357 | Indian River Buffalo | Black | H9 | India |
| 249 | Mf421373.1 Nicra-358 | Indian River Buffalo | Black | H9 | India |
| 250 | Mf421374.1 Nicra-359 | Indian River Buffalo | Black | H9 | India |
| 251 | Mf421375.1 Nicra-360 | Indian River Buffalo | Black | H9 | India |
| 252 | Mf421376.1 Nicra-361 | Indian River Buffalo | Black | H9 | India |
| 253 | Mf421377.1 Nicra-362 | Indian River Buffalo | Black | H9 | India |
| 254 | Mf421378.1 Nicra-363 | Indian River Buffalo | Black | H9 | India |
| 255 | Mf421379.1 Nicra-364 | Indian River Buffalo | Black | H9 | India |
| 256 | Mf421380.1 Nicra-365 | Indian River Buffalo | Black | H9 | India |
| 257 | Mf421381.1 Nicra-366 | Indian River Buffalo | Black | H9 | India |
| 258 | Mf421382.1 Nicra-367 | Indian River Buffalo | Black | H9 | India |
| 259 | Mf421383.1 Nicra-368 | Indian River Buffalo | Black | H9 | India |
| 260 | Mf421384.1 Nicra-369 | Indian River Buffalo | Black | H9 | India |
| 261 | Mf421385.1 Nicra-370 | Indian River Buffalo | Black | H9 | India |
| 262 | Mf421386.1 Nicra-371 | Indian River Buffalo | Black | H9 | India |
| 263 | Mf421387.1 Nicra-372 | Indian River Buffalo | Black | H9 | India |
| 264 | Mf421388.1 Nicra-373 | Indian River Buffalo | Black | H9 | India |
| 265 | Mf421389.1 Nicra-374 | Indian River Buffalo | Black | H9 | India |
| 266 | Mf421390.1 Nicra-375 | Indian River Buffalo | Black | H9 | India |
| 267 | Mf421391.1 Nicra-376 | Indian River Buffalo | Black | H9 | India |
| 268 | Mf421392.1 Nicra-377 | Indian River Buffalo | Black | H9 | India |
| 269 | Mf421393.1 Nicra-378 | Indian River Buffalo | Black | H9 | India |
| 270 | Mf421394.1 Nicra-379 | Indian River Buffalo | Black | H9 | India |
| 271 | Mf421395.1 Nicra-380 | Indian River Buffalo | Black | H9 | India |
| 272 | Mf421396.1 Nicra-381 | Indian River Buffalo | Black | H9 | India |
| 273 | Mf421397.1 Nicra-382 | Indian River Buffalo | Black | H9 | India |
| 274 | Mf421398.1 Nicra-383 | Indian River Buffalo | Black | H9 | India |
| 275 | Mf421399.1 Nicra-384 | Indian River Buffalo | Black | H9 | India |
| 276 | Mf421400.1 Nicra-385 | Indian River Buffalo | Black | H9 | India |
| 277 | Mf421401.1 Nicra-386 | Indian River Buffalo | Black | H9 | India |
| 278 | Mf421402.1 Nicra-387 | Indian River Buffalo | Black | H9 | India |
| 279 | Mf421403.1 Nicra-388 | Indian River Buffalo | Black | H9 | India |
| 280 | Mf421404.1 Nicra-389 | Indian River Buffalo | Black | H9 | India |
| 281 | Mf421405.1 Nicra-390 | Indian River Buffalo | Black | H9 | India |
| 282 | Mf421406.1 Nicra-391 | Indian River Buffalo | Black | H9 | India |
| 283 | Mf421407.1 Nicra-392 | Indian River Buffalo | Black | H9 | India |
| 284 | Mf421408.1 Nicra-393 | Indian River Buffalo | Black | H9 | India |
| 285 | Mf421409.1 Nicra-394 | Indian River Buffalo | Black | H9 | India |
| 286 | Mf421410.1 Nicra-395 | Indian River Buffalo | Black | H9 | India |
| 287 | Mf421411.1 Nicra-396 | Indian River Buffalo | Black | H9 | India |
| 288 | Mf421412.1 Nicra-397 | Indian River Buffalo | Black | H9 | India |
| 289 | Mf421413.1 Nicra-398 | Indian River Buffalo | Black | H9 | India |
| 290 | Mf421414.1 Nicra-399 | Indian River Buffalo | Black | H9 | India |
| 291 | Mf421415.1 Nicra-400 | Indian River Buffalo | Black | H9 | India |
| 292 | Mf421416.1 Nicra-401 | Indian River Buffalo | Black | H9 | India |
| 293 | Mf421417.1 Nicra-402 | Indian River Buffalo | Black | H9 | India |
| 294 | Mf421418.1 Nicra-403 | Indian River Buffalo | Black | H9 | India |
| 295 | Mf421419.1 Nicra-404 | Indian River Buffalo | Black | H9 | India |
| 296 | Mf421422.1 Nicra-407 | Indian River Buffalo | Black | H9 | India |
| 297 | Mf421421.1 Nicra-406 | Indian River Buffalo | Black | H9 | India |
| 298 | Mf421423.1 Nicra-408 | Indian River Buffalo | Black | H9 | India |
| 299 | Mf421424.1 Nicra-409 | Indian River Buffalo | Black | H9 | India |
| 300 | Mf421425.1 Nicra-410 | Indian River Buffalo | Black | H9 | India |
| 301 | Mf421426.1 Nicra-411 | Indian River Buffalo | Black | H9 | India |
| 302 | Mf421427.1 Nicra-412 | Indian River Buffalo | Black | H9 | India |
| 303 | Mf421428.1 Nicra-413 | Indian River Buffalo | Black | H9 | India |
| 304 | Mf421429.1 Nicra-414 | Indian River Buffalo | Black | H9 | India |
| 305 | Mf421430.1 Nicra-415 | Indian River Buffalo | Black | H9 | India |
| 306 | Mf421431.1 Nicra-416 | Indian River Buffalo | Black | H9 | India |
| 307 | Mf421432.1 Nicra-417 | Indian River Buffalo | Black | H9 | India |
| 308 | Mf421433.1 Nicra-418 | Indian River Buffalo | Black | H9 | India |
| 309 | Mf421434.1 Nicra-419 | Indian River Buffalo | Black | H9 | India |
| 310 | Mf421435.1 Nicra-420 | Indian River Buffalo | Black | H9 | India |
| 311 | Mf421436.1 Nicra-421 | Indian River Buffalo | Black | H9 | India |
| 312 | Mf421437.1 Nicra-422 | Indian River Buffalo | Black | H9 | India |
| 313 | Mf421438.1 Nicra-423 | Indian River Buffalo | Black | H9 | India |
| 314 | Mf421439.1 Nicra-424 | Indian River Buffalo | Black | H9 | India |
| 315 | Mf421440.1 Nicra-425 | Indian River Buffalo | Black | H9 | India |
| 316 | Mf421441.1 Nicra-426 | Indian River Buffalo | Black | H9 | India |
| 317 | Mf421442.1 Nicra-427 | Indian River Buffalo | Black | H9 | India |
| 318 | Mf421483.1 Nicra-468 | Indian River Buffalo | Black | H9 | India |
| 319 | Mf421484.1 Nicra-469 | Indian River Buffalo | Black | H9 | India |
| 320 | Mf421485.1 Nicra-470 | Indian River Buffalo | Black | H9 | India |
| 321 | Mf421486.1 Nicra-471 | Indian River Buffalo | Black | H9 | India |
| 322 | Mf421487.1 Nicra-472 | Indian River Buffalo | Black | H9 | India |
| 323 | Mf421488.1 Nicra-473 | Indian River Buffalo | Black | H9 | India |
| 324 | Mf421489.1 Nicra-474 | Indian River Buffalo | Black | H9 | India |
| 325 | Mf421490.1 Nicra-475 | Indian River Buffalo | Black | H9 | India |
| 326 | Mf421491.1 Nicra-476 | Indian River Buffalo | Black | H9 | India |
| 327 | Mf421492.1 Nicra-477 | Indian River Buffalo | Black | H9 | India |
| 328 | Mf421493.1 Nicra-478 | Indian River Buffalo | Black | H9 | India |
| 329 | Mf421494.1 Nicra-479 | Indian River Buffalo | Black | H9 | India |
| 330 | Mf421495.1 Nicra-480 | Indian River Buffalo | Black | H9 | India |
| 331 | Mf421496.1 Nicra-481 | Indian River Buffalo | Black | H9 | India |
| 332 | Mf421497.1 Nicra-482 | Indian River Buffalo | Black | H9 | India |
| 333 | Mf421498.1 Nicra-483 | Indian River Buffalo | Black | H9 | India |
| 334 | Mf421499.1 Nicra-484 | Indian River Buffalo | Black | H9 | India |
| 335 | Mf421500.1 Nicra-485 | Indian River Buffalo | Black | H9 | India |
| 336 | Mf421501.1 Nicra-486 | Indian River Buffalo | Black | H9 | India |
| 337 | Mf421502.1 Nicra-487 | Indian River Buffalo | Black | H9 | India |
| 338 | Mf421503.1 Nicra-488 | Indian River Buffalo | Black | H9 | India |
| 339 | Mf421504.1 Nicra-489 | Indian River Buffalo | Black | H9 | India |
| 340 | Mf421505.1 Nicra-490 | Indian River Buffalo | Black | H9 | India |
| 341 | Mf421506.1 Nicra-491 | Indian River Buffalo | Black | H9 | India |
| 342 | Mf421507.1 Nicra-492 | Indian River Buffalo | Black | H9 | India |
| 343 | Mf421508.1 Nicra-493 | Indian River Buffalo | Black | H9 | India |
| 344 | Mf421509.1 Nicra-494 | Indian River Buffalo | Black | H9 | India |
| 345 | Mf421510.1 Nicra-495 | Indian River Buffalo | Black | H9 | India |
| 346 | Mf421511.1 Nicra-496 | Indian River Buffalo | Black | H9 | India |
| 347 | Mf421512.1 Nicra-497 | Indian River Buffalo | Black | H9 | India |
| 348 | Mf421513.1 Nicra-498 | Indian River Buffalo | Black | H9 | India |
| 349 | Mf421514.1 Nicra-499 | Indian River Buffalo | Black | H9 | India |
| 350 | Mf421515.1 Nicra-500 | Indian River Buffalo | Black | H9 | India |
| 351 | Mf421516.1 Nicra-501 | Indian River Buffalo | Black | H9 | India |
| 352 | Mf421517.1 Nicra-502 | Indian River Buffalo | Black | H9 | India |
| 353 | Mf421518.1 Nicra-503 | Indian River Buffalo | Black | H9 | India |
| 354 | Mf421519.1 Nicra-504 | Indian River Buffalo | Black | H9 | India |
| 355 | Mf421520.1 Nicra-505 | Indian River Buffalo | Black | H9 | India |
| 356 | Mf421521.1 Nicra-506 | Indian River Buffalo | Black | H9 | India |
| 357 | Mf421522.1 Nicra-507 | Indian River Buffalo | Black | H9 | India |
| 358 | Mf421420.1 Nicra-405 | Indian River Buffalo | Black | H9 | India |
| 359 | Gq359888.1 2007029 | Chinese River Buffalo | Not Mentioned | H22 | China |
| 360 | Gu121302.1 Mb37 | Chinese River Buffalo | Black | H22 | China |
| 361 | Gu121303.1 Mb11 | Chinese River Buffalo | Black | H22 | China |
| 362 | Gu121304.1 Mb13 | Chinese River Buffalo | Black | H22 | China |
| 363 | Gu121306.1 Mb17 | Chinese River Buffalo | Black | H22 | China |
| 364 | Gu121307.1 Mb18 | Chinese River Buffalo | Black | H22 | China |
| 365 | Gu121308.1 Mb23 | Chinese River Buffalo | Black | H22 | China |
| 366 | Gu121309.1 Mb24 | Chinese River Buffalo | Black | H22 | China |
| 367 | Gu121310.1 Mb25 | Chinese River Buffalo | Black | H22 | China |
| 368 | Gu121311.1 Mb26 | Chinese River Buffalo | Black | H22 | China |
| 369 | Gu121312.1 Mb32 | Chinese River Buffalo | Black | H22 | China |
| 370 | Gu121313.1 Mb33 | Chinese River Buffalo | Black | H22 | China |
| 371 | Gu121314.1 Mb34 | Chinese River Buffalo | Black | H22 | China |
| 372 | Gu121315.1 Mb36 | Chinese River Buffalo | Black | H22 | China |
| 373 | Gu121315.1 Mb36 (2) | Chinese River Buffalo | Black | H22 | China |
| 374 | Gu121316.1 Mb4 | Chinese River Buffalo | Black | H22 | China |
| 375 | Gu121317.1 Mb6 | Chinese River Buffalo | Black | H22 | China |
| 376 | Gu121318.1 Mb7 | Chinese River Buffalo | Black | H22 | China |
| 377 | Gu121319.1 Mb8 | Chinese River Buffalo | Black | H22 | China |
| 378 | Gu121320.1 Mb9 | Chinese River Buffalo | Black | H22 | China |
| 379 | Gu121321.1 Mb10 | Chinese Swamp Buffalo | Black | H7 | China |
| 380 | Gu121323.1 Mb2 | Chinese Swamp Buffalo | Black | H7 | China |
| 381 | Gu121324.1 Mb3 | Chinese Swamp Buffalo | Black | H7 | China |
| 382 | Gu121325.1 Mb5 | Chinese Swamp Buffalo | Black | H7 | China |
| 383 | Gu121326.1 | Chinese Swamp Buffalo | Black | H7 | China |
| 384 | Gq359888.1 2007029 (2) | Chinese River Buffalo | Black | H22 | China |
| 385 | Km011853 | Indian River Buffalo | Black | H9 | India |
| 386 | Kr859655 | Indian River Buffalo | Black | H9 | India |
| 387 | Km023142 | Indian River Buffalo | Black | H9 | India |
| 388 | Km023143 | Indian River Buffalo | Black | H9 | India |
| 389 | Km023144 | Indian River Buffalo | Black | H9 | India |
| 390 | Km103646.1 Nicra 2 | Indian River Buffalo | Black | H9 | India |
| 391 | Kr363035.1 Nicra-88 | Indian River Buffalo | Black | H9 | India |
| 392 | Kr363049.1 Nicra-89 | Indian River Buffalo | Black | H9 | India |
| 393 | Kr363050.1 Nicra-90 | Indian River Buffalo | Black | H9 | India |
| 394 | Kr363051.1 Nicra-91 | Indian River Buffalo | Black | H9 | India |
| 395 | Kr363052.1 Nicra-92 | Indian River Buffalo | Black | H9 | India |
| 396 | Kr363053.1 Nicra-93 | Indian River Buffalo | Black | H9 | India |
| 397 | Kr363054.1 Nicra-94 | Indian River Buffalo | Black | H9 | India |
| 398 | Kr363055.1 Nicra-95 | Indian River Buffalo | Black | H9 | India |
| 399 | Kr363056.1 Nicra-96 | Indian River Buffalo | Black | H9 | India |
| 400 | Kr363057.1 Nicra-97 | Indian River Buffalo | Black | H9 | India |
| 401 | Kr859648.1 Nicra-99 | Indian River Buffalo | Black | H9 | India |
| 402 | Kr859649.1 Nicra-100 | Indian River Buffalo | Black | H9 | India |
| 403 | Kr859650.1 Nicra-101 | Indian River Buffalo | Black | H9 | India |
| 404 | Kr859651.1 Nicra-102 | Indian River Buffalo | Black | H9 | India |
| 405 | Kr859652.1 Nicra-103 | Indian River Buffalo | Black | H9 | India |
| 406 | Kr859654.1 Nicra-105 | Indian River Buffalo | Black | H9 | India |
| 407 | Kr859654.1 Nicra-105 | Indian River Buffalo | Black | H9 | India |
| 408 | Kr869113.1 Nicra-107 | Indian River Buffalo | Black | H9 | India |
